# Spontaneous deposition of boron oxide on a rhodium nanostructure for selective conversion of syngas to ethanol

**DOI:** 10.1039/d5sc06161j

**Published:** 2025-10-07

**Authors:** Jiale Xiao, Cao Wang, Haotian Meng, Chengtao Wang, Hangjie Li, Yu-Xiao Cheng, Ni Yi, Wentao Yuan, Wei Zhou, Liang Cao, Liang Wang, Feng-Shou Xiao

**Affiliations:** a College of Chemical and Biological Engineering, Department of Chemistry, State Key Laboratory of Chemical Engineering and Low-carbon Technology, Zhejiang University Hangzhou 310058 China ctwang@zju.edu.cn liangcao@zju.edu.cn fsxiao@zju.edu.cn; b Zhejiang Baima Lake Laboratory Co., Ltd Hangzhou 310052 China; c Ningbo Global Innovation Center, Zhejiang University Ningbo 315100 China; d State Key Laboratory of Silicon Materials and Center of Electron Microscopy, School of Materials Science and Engineering, Zhejiang University Hangzhou 310027 China; e Department of Chemistry and Applied Biosciences, ETH Zürich CH-8093 Zurich Switzerland

## Abstract

The selective blocking of specific sites of undesired side reactions on a catalyst nanostructure is important, but challenging. Herein, we show that a boron oxide species could spontaneously and selectively react with the low-coordination sites on Rh nanoparticles, which are responsible for undesired methanation in the conversion of syngas to ethanol. As a result, the boron oxide modified RhMn nanoparticles on a silica support (RhMnB_3.9_/SiO_2_) exhibited oxygenate selectivity as high as 63.9% by methane selectivity reduced to 31.1%, of which 90.1% of the oxygenates are C_2_-oxygenates. Such an oxygenate selectivity outperforms supported RhMn catalysts, which usually exhibit selectivity of higher than 50% for undesired methane. This work offers an alternative route for ethanol production from syngas.

## Abstract 2

The selective blocking of specific sites of undesired side reactions on a catalyst nanostructure is important, but challenging.

## Graphical abstract

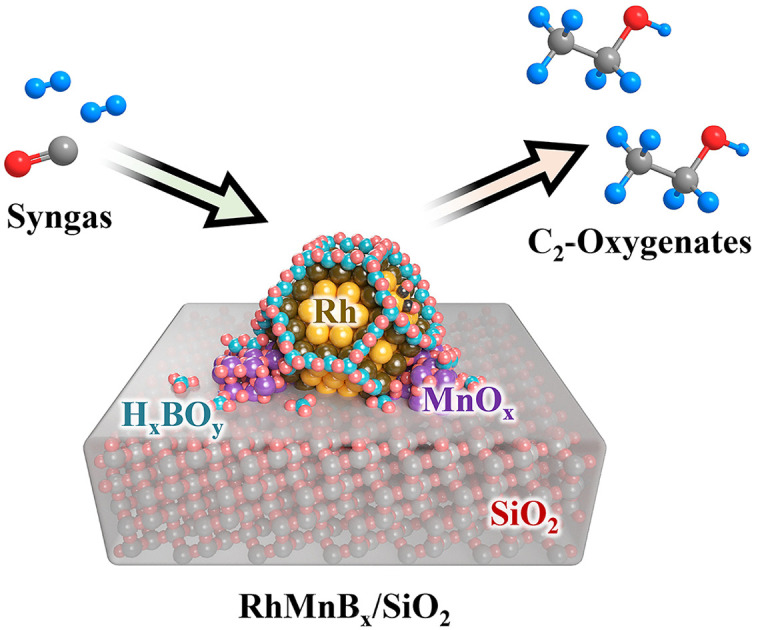

## Introduction

Ethanol is regarded as a crucial platform molecule for the chemical industry to produce valuable chemicals.^1–3^ Current ethanol production methods strongly depend on the fermentation of grains, which competes with human food production. Developing non-grain routes for ethanol production is highly desirable, and multiple routes have been developed using cellulose hydrogenolysis and ethylene hydration.^4–7^ Besides these techniques, there is a trend to produce ethanol from syngas, which is a mixture of CO and H_2_ that can be easily obtained on a large scale from coal, biomass, and natural gas. Since the 1950s, supported Rh nanoparticles with various promoters (*e.g.* Mn,^8–11^ Fe,^12–14^ Cu,^15^ V,^16,17^ Ti,^13,18^ Mo,^19^ Zr,^20,21^ alkali metals,^22,23^ and rare earth metals^17,21,24,25^) have been investigated in the direct conversion of syngas to ethanol. However, the methane selectivity is usually higher than 50%, with C_2_-oxygenate selectivity lower than 40%. The process is still far from meeting the desired selectivity to oxygenate products because of uncontrollable methanation.

Generally, the formation of methane is mainly due to excessive dissociation of carbon–oxygen bonds (C–O) and the subsequent hydrogenation of hydrocarbon intermediates (CH_*x*_).^26–28^ Hindering CO dissociation and weakening the hydrogenation activity of the catalysts is expected to reduce methane selectivity. For example, Fe-promoted Rh could reduce methane formation by increasing the elementary reaction barrier (*CH_3_ + *H → CH_4_ + 2*) from 0.57 eV to 1.21 eV compared to the process using unpromoted Rh, according to DFT calculations.^29^ Very recently, Copéret *et al.* reported the promotional role of Fe in syngas conversion, employing surface organometallic chemistry (SOMC) to prepare well-defined RhFe@SiO_2_ model catalysts.^14^ Compared to the nonpromoted Rh@SiO_2_ yielding methane (selectivity > 90%), the RhFe@SiO_2_ catalyst suppressed the methane selectivity to 41.8%, reaching an ethanol selectivity of 38% among all products at 8.4% CO conversion. In addition, modifying the Rh with alkali metal promoters (such as Li, Na, K, and Cs) should hinder CO dissociation and suppress the methanation reaction. As a result, the selectivity of methane decreases, while that of methanol increases.^30–32^ Note that selectively blocking specific sites responsible for undesired side reactions on the catalyst nanostructure is a promising way to improve the performance, especially for structurally-sensitive processes such as CO dissociation on Rh surfaces.^33,34^ However, control of the catalyst structure at the atomic scale is a challenge.

Herein, we report that methanation can be efficiently hindered in the conversion of syngas to ethanol by partially blocking the low-coordination sites on the Rh nanoparticles for C–O cleavage, giving oxygenate selectivity as high as 63.9% by suppressing the methane selectivity to 31.1%, where 90.1% of the oxygenates are C_2_-oxygenates. This process outperforms the previously tested Rh-based catalysts. The key to this success was the modulation of the Rh-based nanoparticles with boron oxide species. Under the reaction conditions, the boron oxide species could spontaneously migrate and interact with the low-coordination Rh sites, effectively hindering the cleavage of the C–O bond and hydrogenation during the reaction process to improve the ethanol selectivity.

## Results and discussion

### Catalyst synthesis and catalytic performance

**Fig. 1:**
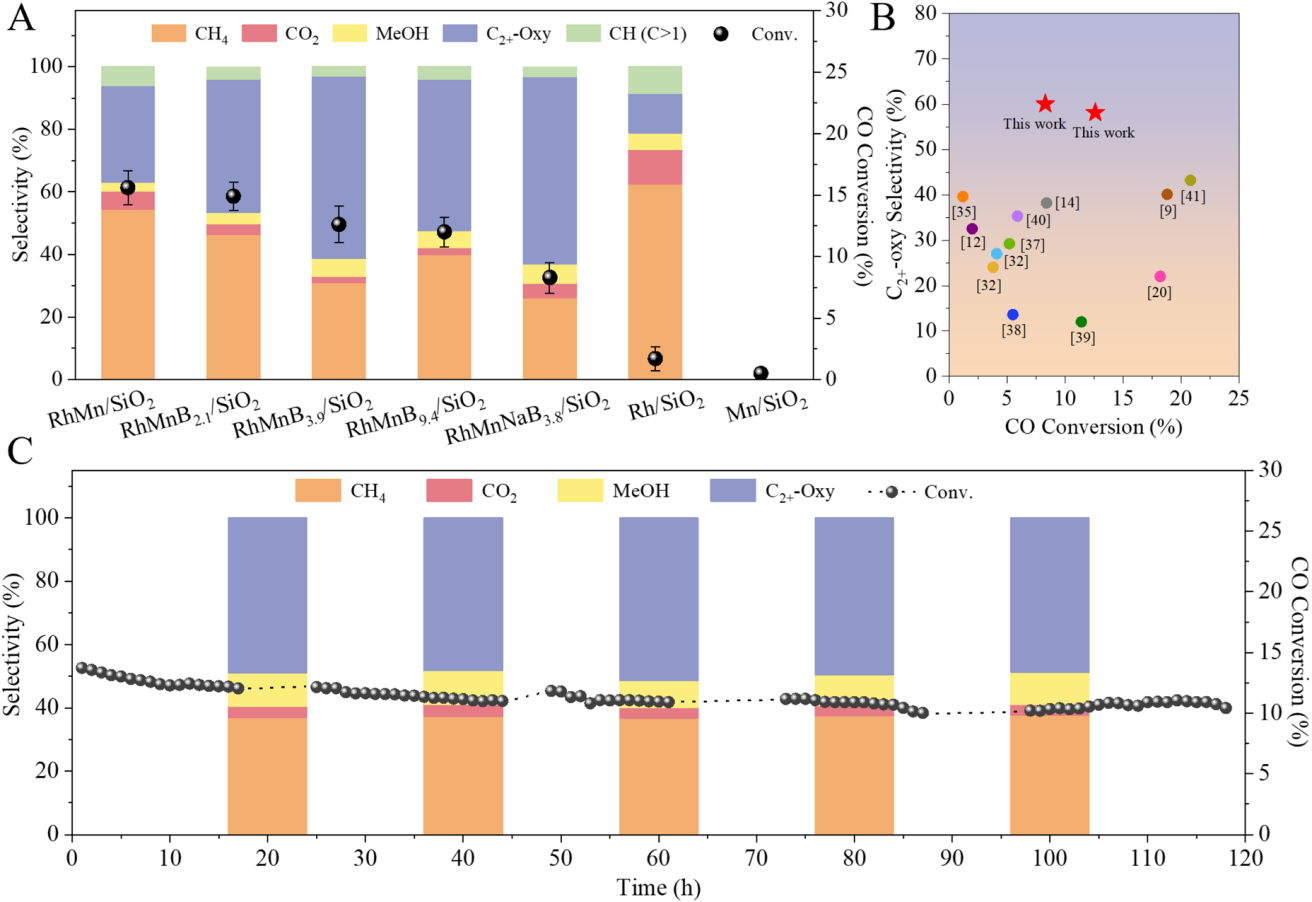
(A) CO conversion and product selectivities over various catalysts. Reaction conditions: 0.5 g of catalyst, H_2_/CO ratio of 2, pressure of 3 MPa, 320 °C, 3360 mL h^−1^ g^−1^. C_2+_-oxygenates (abbr. C_2+_-oxy) is defined as the total oxygenate products except for methanol. (B) Comparison of the catalytic performances of RhMnB_*x*_/SiO_2_ catalysts and Rh-based catalysts reported previously in the conversion of syngas to oxygenates. The details are summarized in Table S4. (C) Data characterizing the durability of the RhMnB_3.9_/SiO_2_ catalysts. Reaction conditions: 0.5 g of catalyst, H_2_/CO ratio of 2, pressure of 3 MPa, 320 °C, 3240 mL h^−1^ g^−1^.

The proof-of-concept experiment employed RhMn nanoparticles supported on silica (RhMn/SiO_2_, Fig. S1 and S2), a classical catalyst for converting syngas to ethanol. Under the reaction conditions (H_2_/CO ratio of 2, pressure of 3 MPa, 320 °C, and 3360 mL g^−1^ h^−1^), the CO conversion was 15.6% with oxygenate selectivity at 33.7% and methane selectivity at 54.4% (Fig. 1A and Table S1). Such a performance was consistent with those of the general RhMn catalysts tested previously.^8,9,35^ The modulation of the RhMn/SiO_2_ catalysts with boric acid was carried out by a physical grinding method (Fig. S3–S7), and the material produced was referred to as RhMnB_*x*_/SiO_2_ (*x* = 2.1, 3.9, and 9.4, where *x* represents the molar ratio of boron and rhodium determined by ICP analysis, Table S2). Linear-scan elemental energy dispersive spectroscopy (EDS) analysis (Fig. S4) and EDS maps (Fig. S5) showed that rhodium, manganese, and boron species were uniformly distributed over the SiO_2_ surface. Compared with RhMn/SiO_2_, the RhMnB_*x*_/SiO_2_ catalysts exhibited much lower methane selectivity with a high selectivity for oxygenate products under the equivalent conditions (Fig. 1A). For example, the RhMnB_2.1_/SiO_2_ catalyst showed a CO conversion of 14.9% with oxygenate selectivity at 46.3%, and the methane selectivity was decreased to 46.4%. The RhMnB_3.9_/SiO_2_ catalyst gave a CO conversion of 12.6% but clearly reduced the methane selectivity to 31.1%. In this case, the selectivity of oxygenate products was 63.9%, 90.1% of which were C_2_-oxygenates, including dominant ethanol and ethyl acetate.

In addition, we changed the boric acid to sodium borohydride to prepare RhMnNaB_3.8_/SiO_2_ catalysts using the same procedures (Fig. S8). The catalytic data showed that the methane selectivity was further decreased to 26.1% with oxygenate product selectivity at 66.0%. Meanwhile, a reduced catalytic activity and a slightly higher methanol selectivity were obtained, which was consistent with the trend reported in the literature.^10,36^ As a blank run, the Rh/SiO_2_ catalyst exhibited very low CO conversion, less than 2%, with methane selectivity at 62.4%, and the Mn/SiO_2_ catalyst could not catalyze the CO hydrogenation. The oxygenate selectivity could be further improved by optimizing the reaction temperatures and the ratios of H_2_/CO (Tables S3 and S4). For example, when the reaction was performed with a lower hydrogen concentration in the feed gas (H_2_/CO at 1), the methane selectivity was decreased to 17.2% with CO conversion at 10.7%, and the oxygenate selectivity was increased to 65.8% (Table S4). It was noteworthy that the selectivity of total oxygenates and alcohols outperformed those of the related catalysts reported previously in syngas conversion (Fig. 1B and Table S5).^9,12,20,32,35,37–41^

**Fig. 2:**
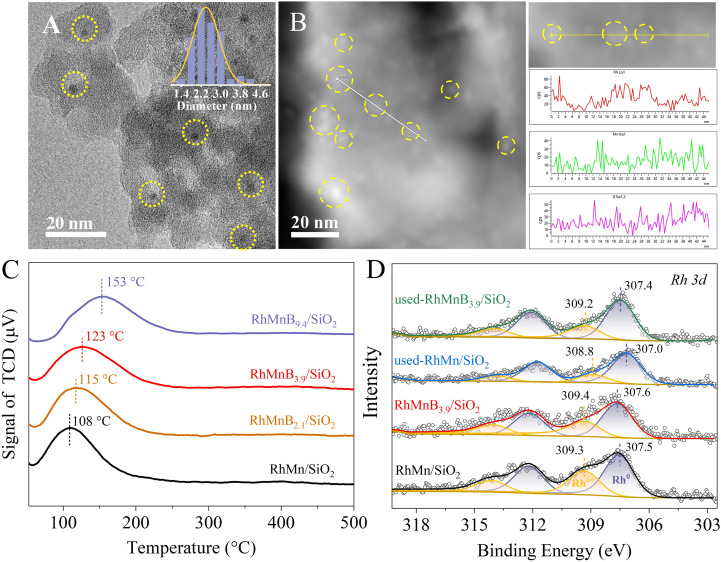
(A) TEM image of the used-RhMnB_3.9_/SiO_2_ after reaction for 118 h. The inset in (A) shows the metal nanoparticle size distribution. (B) STEM image and linear-scan EDS spectra of the used-RhMnB_3.9_/SiO_2_ catalyst. The yellow circles highlight the nanoparticles. (C) H_2_-TPR profiles of various catalysts. (D) Rh 3d XPS spectra of reduced and used RhMn/SiO_2_ and RhMnB_3.9_/SiO_2_ catalysts.

The supported RhMn nanoparticle catalysts usually deactivate within short periods because of rapid Rh nanoparticle sintering under the reaction conditions. Interestingly, the RhMnB_3.9_/SiO_2_ was stable in the test for 118 h at 320 °C, giving an average CO conversion and CH_4_ selectivity of 11.3% and 35.5%, respectively (Fig. 1C). Even after testing for a long time, a CO conversion and CH_4_ selectivity of 10.5% and 35.8% were still obtained, indicating an almost unchanged performance compared with the initial reaction. As shown in Fig. S9, the used RhMn/SiO_2_ catalyst (time on steam at 30 h) showed that the Rh NPs were partially sintered with a wide size distribution of 2–11 nm (average size of 3.5 nm), while the used RhMnB_3.9_/SiO_2_ catalyst showed a narrow size distribution of 1.4–3.8 nm (average size of 2.3 nm). Even after 118 h in the test, the used RhMnB_3.9_/SiO_2_ catalyst still showed a relatively narrow size distribution of nanoparticles of 1.4–4.2 nm with an average size of 2.4 nm (Fig. 2A), very similar to those of the fresh catalyst, confirming the sinter resistance of the Rh nanoparticles. It is worth noting that very few boron species on the used RhMnB_3.9_/SiO_2_ catalyst were leached during the reaction, as evidenced by the ICP results (Table S2). In addition, the EDS maps (Fig. S10) and EDS line scans (Fig. 2B) showed that the signals of rhodium, manganese, and boron on the used RhMnB_3.9_/SiO_2_ catalyst were also similar to those of the fresh catalyst (Fig. S4 and S5).

### Modification by boron oxide species

Considering that RhMn/SiO_2_ and RhMnB_*x*_/SiO_2_ have similar nanoparticle sizes and the same SiO_2_ support, the different catalytic performances should be due to the modification by boron oxide species. Hydrogen temperature programmed reduction (H_2_-TPR) was performed to characterize the reducibility of catalysts (Fig. 2C). The H_2_ consumption peaks moved to higher temperatures gradually with increased boron amount. The boric-free RhMn/SiO_2_ catalyst displayed a H_2_ consumption peak centered at 108 °C, while the reduction temperatures of the RhMnB_2.1_/SiO_2_, RhMnB_3.9_/SiO_2_, and RhMnB_9.4_/SiO_2_ catalysts were moved from 115 °C to 153 °C, indicating the suppression of reduction. This phenomenon was probably due to the chemical interaction between boric oxide and the Rh species, which enhanced the resistance to reduction of the Rh species.

To characterize the electronic states of Rh species, X-ray photoelectron spectroscopy (XPS) was used to test the Rh 3d binding energy values (BEs) of the RhMn/SiO_2_ and RhMnB_3.9_/SiO_2_ catalysts (Fig. 2D). The deconvolutions of the Rh 3d_5/2_ peaks at 307.0–307.6 eV and 308.8–309.4 eV are attributed to Rh^0^ and Rh^δ+^, respectively.^11,35,42^ Before the reaction, the peak area ratios of Rh^δ+^/Rh^0^ were 0.61 and 0.67 for RhMn/SiO_2_ and RhMnB_3.9_/SiO_2_ (Table S6). After reaction for 30 h in the syngas (H_2_/CO ratio at 2, pressure at 3 MPa, 320 °C, and 3360 mL g^−1^ h^−1^), the ratio of Rh^δ+^/Rh^0^ changed to 0.25 and 0.37, confirming the Rh^δ+^ species on the RhMnB_3.9_/SiO_2_ were more stable than those on the RhMn/SiO_2_ during the reaction.

The XPS spectra of Mn 2p and B 1s were also studied, as shown in Fig. S11. The BEs of the Mn 2p on the RhMn/SiO_2_ and RhMnB_3.9_/SiO_2_ catalysts were observed at ∼642.4 eV, indicating the presence of manganese oxides in both catalysts.^11,43,44^ There were almost no changes for Mn 2p peaks before and after reaction (Fig. S11A), indicating that the manganese remained oxidized. A widely accepted perspective suggests that the presence of amorphous MnO_*x*_ surrounding the Rh species is crucial in promoting CO-adsorption and dissociation, thereby significantly enhancing reaction rates. Furthermore, the generated interfacial sites (Rh^δ+^–O–Mn) benefit CO insertion to improve the oxygenate selectivities,^11,45,46^ which was also confirmed by our catalytic data in Table S1 (entries 1 and 2). Fig. S11B shows that the signals of B 1s were observed at 193.2 eV for the RhMnB_3.9_/SiO_2_ and used-RhMnB_3.9_/SiO_2_, suggesting the chemical state of boron was +3 in the form of B_2_O_3_.^47–49^ However, the binding energy of B 1s was nearly unchanged before and after the reaction. One possible reason is that electron transfer between Rh and B could occur through O atoms (Fig. S11C), because the boron is mainly present as boron oxide species.^47,50^ Another possible reason is lower detection sensitivity for boron (as a light element), leading to the nearly unchanged binding energy of B 1s.

The chemical state of the Rh species was further studied using X-ray absorption near-edge structure (XANES) measurement. Fig. S12A shows XANES spectra of the RhMn/SiO_2_ and RhMnB_3.9_/SiO_2_ with high pre-edge energy, indicating positively charged Rh species. After reaction, the pre-edge energy shifted from the Rh_2_O_3_ feature to the Rh metallic feature, suggesting that the Rh^δ+^ species were partially reduced by syngas.^10^ Notably, the used-RhMnB_3.9_/SiO_2_ exhibited higher pre-edge energy than the used-RhMn/SiO_2_, implying that the Rh species on the used-RhMnB_3.9_/SiO_2_ were more positively charged than those on the used-RhMn/SiO_2_, which was in good agreement with the XPS results. Fig. S12B and Table S7 show extended X-ray absorption fine structure (EXAFS) data of the Rh species on the RhMn/SiO_2_ and RhMnB_3.9_/SiO_2_ before and after reaction. All the catalysts showed the different peaks assigned to Rh–O and Rh–Rh coordination. After reaction, the Rh–Rh signals were enhanced due to the reduction of the Rh species by the syngas. The used-RhMnB_3.9_/SiO_2_ catalyst exhibited a higher ratio of Rh–O/Rh–Rh signal than that of the used-RhMn/SiO_2_ catalyst, suggesting the Rh species were more positive on the used-RhMnB_3.9_/SiO_2_ catalyst. These results suggest the efficient modification of boron oxide species to Rh nanoparticles on the RhMnB_3.9_/SiO_2_ catalyst.

### Spontaneous dispersion of boron species during the reaction process

CO-temperature programmed desorption (CO-TPD) was performed to investigate the CO-adsorption behavior on the RhMn/SiO_2_ and RhMnB_3.9_/SiO_2_ catalyst before and after reaction (Fig. S13). Two CO-desorption peaks located in the ranges 80–180 °C and 250–350 °C were observed for all the catalysts. According to previous studies, the thermal stability of adsorbed CO is in the following order: linear CO < geminal CO < bridged CO.^51,52^ Therefore, the low temperature desorption peak should be assigned to the linear CO species, while the high temperature desorption peak corresponds to the geminal adsorbed CO species and bridged CO species. For the RhMn/SiO_2_ catalyst, the desorption signals of CO were slightly weakened after reaction. In contrast, the CO-desorption peaks were significantly reduced on the RhMnB_3.9_/SiO_2_ after reaction. This phenomenon implies the optimization of the structure of the RhMnB_3.9_/SiO_2_ catalyst during the reaction, leading to a lower capacity for CO-adsorption.

**Fig. 3:**
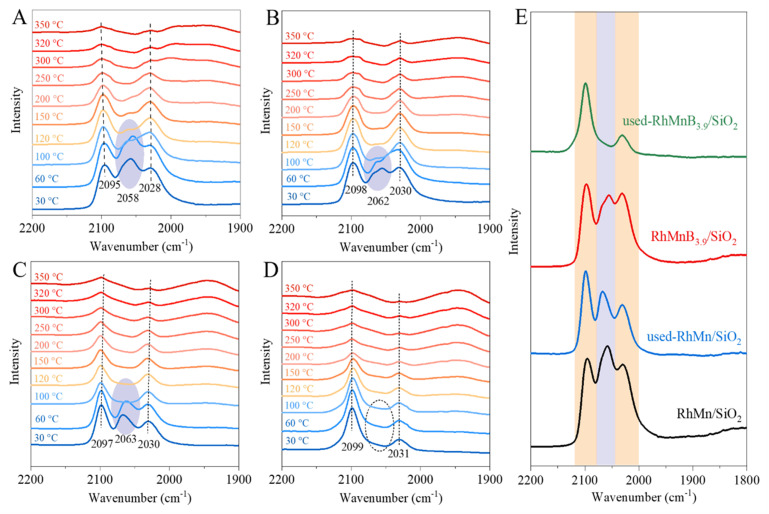
CO-desorption *in situ* IR spectra of (A) RhMn/SiO_2_, (B) RhMnB_3.9_/SiO_2_, (C) used-RhMn/SiO_2_ and (D) used-RhMnB_3.9_/SiO_2_ catalysts at different temperatures. (E) CO-desorption *in situ* IR spectra at 30 °C. The data is collected from (A–D).

CO-adsorption Fourier Transform Infrared Spectroscopy (FTIR) was investigated to study the states of CO-adsorption on the Rh species over the RhMn/SiO_2_ and RhMnB_3.9_/SiO_2_ catalysts before and after the reaction (Fig. 3). The CO chemisorption bands at 2095–2098, 2058–2062, and 2028–2030 cm^−1^ were assigned to the symmetrical stretching of gem-dicarbonyl CO-adsorption on Rh^δ+^-(CO)_2_ or the linear CO-adsorption on Rh^δ+^-(CO), linear CO-adsorption on Rh^0^-(CO), and asymmetrical stretching of gem-dicarbonyl CO-adsorption on Rh^δ+^-(CO)_2_, respectively.^11,23,53^ Upon increasing the temperature of the sample cell from 30 °C to 350 °C, the signals of the gem-dicarbonyl CO and linear CO-adsorption bands gradually decreased. Specifically, the band of Rh^0^-(CO) for the RhMn/SiO_2_ gradually decreased from 30 °C to 120 °C, and almost disappeared when the temperature was higher than 120 °C (Fig. 3A). Similar phenomena occurred for the RhMnB_3.9_/SiO_2_, where the band of Rh^0^-(CO) disappeared at 100 °C (Fig. 3B). Interestingly, significant changes were observed in the band of Rh^0^-(CO) for the used catalysts. Notably, the Rh^0^-(CO) band disappeared at 100 °C for the used RhMn/SiO_2_ catalyst (Fig. 3C), while the Rh^0^-(CO) band even disappeared at 30 °C for the used RhMnB_3.9_/SiO_2_ (Fig. 3D). According to previous studies, the types of CO adsorbed on Rh nanoparticles are very sensitive to the structure and chemical environment of the Rh surfaces.^34,54–57^ Based on the catalytic data and structural characterization results, we speculate that the boron oxide species on the RhMnB_3.9_/SiO_2_ might be mobile during the reaction, and efficiently modify the Rh nanoparticles, thus weakening the linear CO-adsorption on the Rh nanoparticles.

### DFT studies

**Fig. 4:**
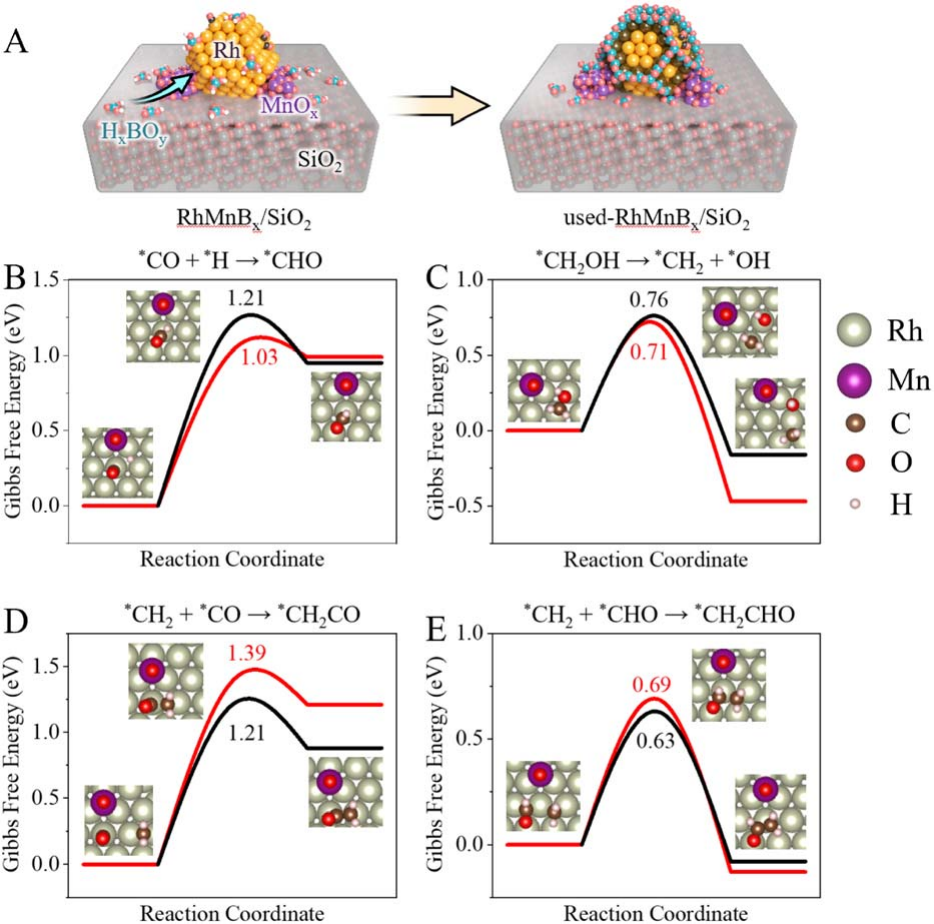
(A) Models showing the optimization of the RhMnB_*x*_/SiO_2_ catalyst structure during the reaction by spontaneous deposition of boron oxide species. (B–E) Gibbs free energy diagrams and configurations for four intermediate steps on Mn_1_O_1_ supported on the Rh (111) facet (black line) and (221) facet (red line) at 595 K. The insets in (B–E) show the configurations of the initial state (IS), transition state (TS) and final state (FS) on Rh (111).

To understand the reaction process and thereby rationalize the distinct catalytic performances of the RhMnB_*x*_/SiO_2_ compared with the commonly supported RhMn/SiO_2_ catalysts, we performed density functional theory (DFT).^58,59^ Fig. S14 and Table S8 showed that the (111), (221), (322), and (100) facets constitute the majority of Rh surfaces in Wulff construction.^60,61^ The monomer Mn_1_O_1_ supported on the Rh slab has been proved to be stable^11^ and was chosen to represent the Rh–MnO_*x*_ model structure in subsequent calculations (Fig. S15–S18). We first investigated the adsorption energies (Δ*E*) of boron oxide on (100) and (111) facets (*i.e.* low index facets) and (221) and (322) facets (*i.e.* high index facets) (Fig. S19). The Δ*E* of the (221), (100), and (111) facets relative to that of the (322) facet are summarized in Table S9. The results showed that the Δ*E* values of boron oxides on the (221), (100) and (111) facets are 0.15, 0.20, and 0.36 eV higher than that of the (322) facet, indicating that boron oxide was much more inclined to adsorb on low-coordinated Rh sites (*i.e.*, step sites on (322) and (221) facets) than high-coordinated Rh sites (*i.e.*, terrace sites on (100) and (111) facets, Fig. 4A). Considering that CO molecules also preferred to adsorb on the low-coordinated Rh sites over high-coordinated Rh sites (Table S9), we suggest that the modulation of the Rh-based nanoparticles with boron oxide species could efficiently influence the adsorption and activation of CO molecules.

We further calculated the Gibbs free energy activation barriers (Δ*G*_a_) of key intermediate steps to investigate the syngas conversion process over RhMn-based catalysts. Two facets, (111) and (221), were employed to represent the terrace sites and step sites on Rh nanoparticles for the following calculations (Fig. S20–S23). Generally, methane is primarily formed *via* the cleavage of the C–O bond followed by subsequent hydrogenation of the *CH_*x*_ intermediates, whereas the oxygenates are mainly generated by the coupling reaction of *CH_*x*_ and the undissociated CO-derived intermediates, such as *CHO or *CO species. Therefore, the cleavage of the C–O bond and the further transformation of the *CH_*x*_ intermediates are the key intermediate steps (Fig. S24). With regard to the cleavage of the C–O bond, our calculation results indicate that hydrogen-assisted dissociation is preferred for the direct dissociation on both Rh (111) and (221) facets (entries 1–6 in Table S10), which is in good agreement with the previous studies.^33,62^ More importantly, the hydrogenation energy barriers of *CO species on the Rh (221) facet are generally lower than that on the Rh (111) facet in various possible hydrogenation steps, especially in the first hydrogenation step (the Δ*G*_a_ on the Rh (221) facet is 0.18 eV lower than that on the Rh (111) facet, Fig. 4B), which means that the subsequent cleavage of the C–O bond is more favourable.^63,64^

According to the literature, *CO is predominantly transformed to *CH_2_OH and *CH_3_O species after three hydrogenation steps, and then *CH_2_ and *CH_3_ are produced through C–O dissociation.^65,66^ Therefore, *CH_2_OH → *CH_2_ + *OH and *CH_3_O → *CH_3_ + *O were chosen as representative C–O dissociation steps in this work to investigate the cleavage of the C–O bond on the Rh (111) and (221) facets. Table S10 shows that the Δ*G*_a_ of *CH_2_OH → *CH_2_ + *OH was lower on the Rh (221) facet than on the Rh (111) facet (0.71 eV and 0.76 eV, entry 7), while the Δ*G*_a_ of the *CH_3_O → *CH_3_ + *O step on the Rh (221) facet was 0.17 eV lower than that on the Rh (111) facet (entry 8), suggesting that both *CH_2_OH → *CH_2_ + *OH and *CH_3_O → *CH_3_ + *O steps were more likely to occur on the Rh (221) facet (Fig. 4C). These results showed that the low-coordinated Rh sites (*i.e.* step sites on high index facets) were favourable for the *CO hydrogenation and C–O bond cleavage.

Although experimentally investigating the CH_*x*_O decomposition is challenging, the performance of different catalysts for the C–O cleavage of *CH_3_O species during methanol decomposition to methane can be evaluated using a well-designed strategy, because methanol readily loses the hydroxyl hydrogen on the catalyst surface. Therefore, we performed temperature-programmed surface reaction experiments of methanol (MeOH-TPSR) in a fixed-bed glass reactor connected to a mass spectrometry instrument. As shown in Fig. S25, the signal of methanol was decreased at about 200 °C, and the signals of H_2_ and CO centered at about 395 °C on the RhMn/SiO_2_ catalyst, indicating that methanol decomposition and dehydrogenation of the *CH_3_O species occurred. In addition, the centers of the water and methane signals were detected at about 470 °C and 510 °C, which is attributed to the C–O cleavage and deep hydrogenation on the RhMn/SiO_2_ catalysts. In contrast, the temperature of methanol decomposition on the RhMnB_3.9_/SiO_2_ catalyst was increased to 230 °C, and the CO signal and H_2_ signal center were moved to 500 °C. More importantly, almost no water or methane signals were observed on the RhMnB_3.9_/SiO_2_ catalyst. These results suggest the weakened ability of the RhMnB_3.9_/SiO_2_ catalyst in the cleavage of the C–O bond, which is consistent with the theoretical calculation.

As for the CH_*x*_ intermediates produced by CO dissociation, one reaction path is to generate methane and higher hydrocarbons by further self-coupling and hydrogenation, and the other path is coupling with the non-dissociated CO-derived species (CO/*CH_*x*_O) to form oxygenates (Fig. S23). We calculated the energy barriers of the CH_*x*_ hydrogenation step (*CH_2_ + *H → *CH_3_) and the coupling steps (*CH_2_ + *CO → *CH_2_CO, and *CH_2_ + *CHO → *CH_2_CHO) on the Rh (111) and (221) facets (Table S10, entry 9–11). The data showed that the Δ*G*_a_ of the *CH_2_ + *H → *CH_3_ step on the Rh (111) facet was 0.09 eV higher than that on the Rh (221) facet, whereas the Δ*G*_a_ values of the *CH_2_ + *CO → *CH_2_CO and *CH_2_ + *CHO → *CH_2_CHO steps on the Rh (111) facet were 0.18 eV and 0.06 eV lower than those on the Rh (221) facet (Fig. 4D and E), indicating that the hydrogenation step tended to proceed with the Rh (221) facet, and the coupling step on the Rh (111) facet was more favourable to produce C_2_-oxygenates.

These results suggest that C–O bond cleavage and hydrogenation preferentially occur on low-coordinated Rh sites with high index facets, while C–C coupling favors high-coordinated Rh sites with low index facets. Notably, boron oxide species selectively deposit on low-coordinated Rh sites during reaction, effectively suppressing C_1_ by-products and enhancing C_2_-oxygenate selectivity.

## Conclusions

In summary, we reported a simple modification for a metal nanostructure achieved by physically grinding RhMn catalysts with boron promoters. The structural characterizations and DFT calculations demonstrate that the boron oxide species could spontaneously and selectively block the low-coordination sites on the Rh nanoparticles, which are active sites for C–O cleavage and CH_*x*_ hydrogenation. Owing to this feature, the undesired methanation in the conversion of syngas to ethanol was suppressed, and the oxygenate selectivity was increased to as high as 63.9%. This work provides a new route for preparing efficient heterogeneous catalysts for the selective conversion of syngas to ethanol.

## Author contributions

Jiale Xiao – data curation, formal analysis, investigation. Cao Wang – data curation, formal analysis, software. Haotian Meng – data curation, formal analysis, validation. Chengtao Wang – conceptualization, formal analysis, investigation, resources, supervision, funding acquisition, writing – original draft, writing – review & editing. Hangjie Li – formal analysis, funding acquisition. Yu-Xiao Cheng – data curation, formal analysis. Ni Yi – data curation, formal analysis. Wentao Yuan – formal analysis. Wei Zhou – formal analysis. Liang Cao – formal analysis, software, supervision, funding acquisition, writing – original draft, writing – review & editing. Liang Wang – conceptualization, formal analysis, writing – original draft. Feng-Shou Xiao – conceptualization, formal analysis, writing – original draft, writing – review & editing.

## Conflicts of interest

The authors declare no conflict of interest.

## Supplementary Material

SC-OLF-D5SC06161J-s001

## Data Availability

The data that support the findings of this study are available in the article or in the supplementary information (SI). Supplementary information: XRD patterns, N_2_ isotherm, SEM images, TEM images, NH_3_-TPD profiles, CO-TPD profiles, H_2_-TPR profiles, CO-adsorption FTIR spectra, MeOH-TPSR data and DFT results. See DOI: https://doi.org/10.1039/d5sc06161j.
