# Supplementary material for: Spontaneous deposition of boron oxide on a rhodium nanostructure for selective conversion of syngas to ethanol
Source: Chem Sci. 2025 Oct 7;16(46):22002–9. doi: 10.1039/d5sc06161j (PMC12536646; doi:10.1039/d5sc06161j)
Supplement: SC-016-D5SC06161J-s001 [file SC-016-D5SC06161J-s001.pdf]

Supplementary Information (SI) for Chemical Science.
This journal is © The Royal Society of Chemistry 2025

Supplementary information (SI)

Spontaneous deposition of boron oxide on rhodium nanostructure for selective conversion of syngas to ethanol

Jiale Xiao,[a,c,#] Cao Wang,[a,#] Haotian Meng,[a,c,#] Chengtao Wang,[a,b,c,*] Hangjie Li,[a] Yu-Xiao Cheng,[a] Ni Yi,[a] Wentao Yuan,[d] Wei Zhou,[e] Liang Cao,[a,*] Liang Wang,[a,b,c] Feng-Shou Xiao[a,b,c,*]

[a] College of Chemical and Biological Engineering, Department of Chemistry, and State Key Laboratory of Chemical Engineering and Low-carbon Technology, Zhejiang University, Hangzhou, 310058, China.

[b] Zhejiang Baima Lake Laboratory Co., Ltd., Hangzhou, 310052, China.

[c] Ningbo Global Innovation Center, Zhejiang University, Ningbo, 315100, China.

[d] State Key Laboratory of Silicon Materials and Center of Electron Microscopy, School of Materials Science and Engineering, Zhejiang University, Hangzhou, 310027 China.

[e] Department of Chemistry and Applied Biosciences, ETH Zürich, CH-8093 Zurich, Switzerland.

*Corresponding author: Chengtao Wang (ctwang@zju.edu.cn); Liang Cao (liangcao@zju.edu.cn); Feng-Shou Xiao (fsxiao@zju.edu.cn)

Materials

Boric acid (>99%), sodium borohydride (97%), ammonium hydroxide (AR, 25%~28%), acetaldehyde (99%), n-butanol (99.5%, GC), methyl formate (99%, GC), methyl acetate (99%, GC), ethyl acetate (99.7%, GC), SiO_2 (AR) and manganese acetate tetrahydrate were obtained from Aladdin Chemical Reagent Company. H_2O_2 (AR, 30%), methanol (99.5%, AR), 1-propanol (99.5%, AR), ethylene glycol (99.5%, AR), dimethyl sulfoxide (98%, CP) and HCl (Ar, 36~38%) were obtained from Sinopharm Chemical Reagent Co. Ltd. Ethanol (AR, 99.7%) was obtained from Shanghai Lingfeng Chemical Reagent Co., Ltd. Urea (99.5%) was obtained from J&K China Chemical Ltd. Rhodium chloride hydrate (Rh 39%) was obtained from Beijing HWRK Chem Co., Ltd. H_2, 90% CO/Ar, He, N_2 were supplied by Hangzhou Jingong special gas Co. Ltd.

Catalyst preparation

Synthesis of RhMn/SiO_2, Rh/SiO_2 and Mn/SiO_2 catalysts. As a typical run for the synthesis of RhMn/SiO_2 catalyst, 2.0 g of SiO_2 and 3.35 g of urea were added into 100 mL of water under stirring, then 89.2 mg of manganese acetate tetrahydrate and 2.63 mL $RhCl_3 \cdot 3H_2O$ solution (Rh concentration at 7.61 mg/mL) were added. After stirring at 80 °C for 4 h, the liquor was cooled to room temperature, and the RhMn/SiO_2 sample was obtained by filtering, washing with distilled water, and drying at 100 °C for 12 h. The synthesis procedures of Rh/SiO_2 and Mn/SiO_2 catalysts were identical to those mentioned above for the synthesis of RhMn/SiO_2 catalyst except using 2.63 mL $RhCl_3 \cdot 3H_2O$ solution (Rh concentration at 7.61 mg/mL) and 89.2 mg of manganese acetate tetrahydrate as metal precursor, respectively.

Synthesis of RhMnB_x/SiO_2 modified by boron acid. The RhMn/SiO_2 modified by a boron acid was synthesized from a physical grinding method. As a typical run, 1.0 g of RhMn/SiO_2 and 18.0 mg of boron acid were mixed and fully ground at room temperature for 30 min, then the mixture was calcinated at 400 °C for 4 h in air, and reduced at 300 °C in 10% H_2/Ar for 2 h, the RhMn/SiO_2 modified by boron catalyst was obtained, donated as RhMn$B_{3.9}$/SiO_2 (the mole ratio of B/Rh at 3.9, measured by ICP). For comparison, the RhMn/SiO_2 modified by different amounts of boron acid (the mole ratio of B/Rh at 2.1 and 9.4, measured by ICP) were synthesized by similar procedures except using 6.0 and 42.0 mg of boron acid.

Synthesis of RhMnNa$B_{3.8}$/SiO_2 modified by sodium borohydride. The synthesis of RhMnNa$B_{3.8}$/SiO_2 was prepared by similar procedures as the synthesis of RhMnB_x/SiO_2, except using 11.0 mg of sodium borohydride.

Catalyst characterization

The catalyst after reaction was collected from the reactor and sealed up (<24 h) before the characterization. Powder X-ray diffraction (XRD) patterns were collected on a Rigaku D/MAX

2550 diffractometer with Cu Kα radiation (λ=1.5418Å). The metal content was determined by inductively coupled plasma analysis (ICP, PerkinElmer NexION 300X and Agilent Technologies 7800 ICP-MS). Transmission electron microscopy (TEM) images, EDS mapping and linear-scan EDS spectra were performed on a JEM-2100F electron microscopy (JEOL, Japan) with an acceleration voltage of 200 kV. HAADF-STEM images and EDS mapping were performed on an FEI Titan G2 80–200 ChemiSTEM (200 kV) scanning transmission electron microscope, equipped with a spherical corrector providing a spatial resolution of ≈0.8 Å. XANES and EXAFS data of the Rh *K*-edge were collected in a transmission mode on beamline BL14W1 at Shanghai Synchrotron Radiation Facility (SSRF).

CO temperature programmed desorption (CO-TPD) was performed on a BELCAT II instrument. Typically, the sample was pretreated with Ar gas flow at 400 °C for 2 h. Then, the Ar flow was switched off and a CO/Ar (5 vol% CO) gas mixture was introduced for CO adsorption. After adsorption at 40 °C for 1 h, the gas feed was switched to Ar at 40 °C for 0.5 h. CO-TPD profiles were collected in flowing Ar by raising the temperature from 40 to 600 °C with a ramping rate of 10 °C/min.

Hydrogen temperature programmed reduction (H_2-TPR) was performed on a catalyst analyzer BELCAT II instrument. Prior to the measurement, 100 mg catalyst was pretreated in a quartz U-tube reactor at 300 °C for 1 h under an Ar gas flow to drive off physically adsorbed impurities. After the catalyst cooled down to 50 °C for a while, 10%H_2/Ar was introduced at a flow rate of 30 mL/min, and then the temperature was ramped linearly (10 °C/min) from 50 °C to 500 °C. Hydrogen consumption was simultaneously monitored by a thermal conductivity detector (TCD).

CO-adsorption FTIR spectra were recorded using a Thermo Nicolet iS10 spectrometer with a mercury-cadmium-telluride detector (MCT detector). As the typical run for fresh catalysts, 50 mg of the sample was localized in the crucible and pre-reduced with 10% H_2/N_2 at 400 °C for 1 h (flow rate at 30 mL/min). Then, the temperature was cooled down to 30 °C, and a flow of 30 mL/min pure He was introduced into the system at 30 °C to collect background data. Thereafter, the sample was treated with a flow of 30 mL/min 2% CO/He for 0.5 h to ensure the saturated CO adsorption, followed by switching to pure He to remove any physically adsorbed CO, and the spectra were recorded when the signals were unchanged. Sequentially, the sample cell was heated to 60, 100, 120, 150, 200, 250, 300, 320, 350 °C with a heating rate of 5 °C/min, and kept constant for 10 min at every temperature point to record the spectra. For the used catalysts, the characterization was performed by similar procedures except that the pre-reduced step was followed by pretreatment with syngas (H_2/CO at 1, 0.1 MPa) at 320 °C for 12 h (flow rate at 20 mL/min).

Temperature programmed surface reaction of methanol (MeOH-TPSR) was performed in a fixed-bed glass reactor connected to a mass spectrum instrument (SRD200M, TILON GRP TECHNOLOGY LIMITED). As a typical run for the fresh catalysts, 20 mg of sample was localized in the bottom of the U type reactor tube and pre-reduced with 20% H_2/He at 400 °C for 1 h (flow rate at 20 mL/min), followed by pretreatment with syngas (H_2/CO at 1, 0.1 MPa) at 320 °C for 12 h (flow rate at 20 mL/min). Then, the temperature was cooled down to 50 °C and the gas was switched to pure He gas (flow rate at 15 mL/min) for 10 min to replace the atmosphere in the pipes. After that, the methanol bubbling with a flowing pure He gas was introduced into the system for 50 min to stabilize the baseline of the mass spectra. MeOH-TPSR profiles were collected by raising the temperature from 50 to 650 °C with a ramping rate of 10 °C/min.

Computational Methods

Details of DFT calculations

All the density functional theory (DFT)[1,2] calculations were carried out using projector-augmented plane-wave (PAW) method[3] and the revised Perdew-Burke-Ernzerhof (RPBE) exchange-correlation functional at the generalized gradient approximation (GGA) level[4] as implemented in the Vienna Ab-initio Simulation Package (VASP).[5] We set the plane-wave cut-off energy to 434.431 eV and sampled the Brillouin zone using 3×3×1 Gamma-centered k-point mesh.[6] The convergence criteria for electronic energy and force were 10^{-6} eV and 0.03 eV/Å, respectively. Spin polarization was considered in the calculations and second-order Methfessel-Paxton smearing[7] with a width of 0.2 eV was used to set partial occupancies. The lattice parameter used for fcc Rh was 3.85 Å. We chose the 4×4 Rh slabs containing 4 atomic layers with the bottom two layers fixed to their bulk positions and the vacuum layer was set to 20 Å. The combination of CI-NEB[8] and Dimer[9] methods was utilized for the transition state searching. We performed the frequency analysis to ensure that we found the exact transition states. To be consistent with the experimental temperature, the Gibbs free energy corrections at 595 K were handled by using VASPKIT.[10] Structure visualization was implemented by VESTA.[11]

Wulff construction of Rh nanoparticles

The surface energies of different Rh facets were performed using DFT calculations with the PBEsol exchange correlation functional.[12] All Rh slabs have 4-layer atoms at least and are separated by 18 Å of vacuum space in the direction perpendicular to the surface plane which is enough to make sure no interactions between two slabs. The surface energy is defined as:

$$E_{surf} = \frac{E_{slab}^{DFT} - \frac{N_{slab}}{N_{bulk}} * E_{bulk}^{DFT}}{2 * A}$$

where E_{slab}^{DFT} is the DFT energy of per unit cell of a Rh slab with all atoms allowed to relax in all directions and lattice parameters in the direction parallel to the surface fixed to be the same as bulk lattice parameter, E_{bulk}^{DFT} is the energy of per unit cell of the Rh bulk crystal, N_{slab} is the number of atoms per unit cell of the slab, N_{bulk} is the number of atoms per unit cell of the bulk crystal, and A is the surface area of one side of slab. For each slab we have two surfaces, which is reflected by the number 2 in the denominator. The surface energies for eight facets were calculated, including (100), (110), (111), (211), (221), (310), (311) and (322). The equilibrium morphology of Rh nanoparticle (Figure S14) was constructed by Wulff's method,[13,14] using a WulffPack python package.[15] The percentages of the surface areas covered by these facets are shown in Table S8.

MnO supported on Rh particle surfaces

Recently, Nørskov et al. constructed the monomer and stripe MnO supported Rh slab models which well demonstrated how Rh-MnO interface sites enhance the C_{2+}-oxygenates production.[16] Consequently, we adopted the Mn_1O_1, Mn_3O_3 and Mn_4O_4 supported Rh slab models in this work, which are denoted as Rh/Mn_1O_1, Rh/Mn_3O_3, and Rh/Mn_4O_4, respectively. According to Wulff construction of Rh, (111), (221) and (322) are the main facets and both (221) and (322) are high-index facets that would exhibit similar catalytic structure-property relationships. Therefore, (111) and (221) facets were chosen as two most representative facets to investigate. For Rh/Mn_1O_1, the hcp site on (111) facet and fcc site on (221) facet was identified as the most stable Mn_1O_1 supported sites, respectively (Figures S15-S17). For Rh/Mn_3O_3 and Rh/Mn_4O_4, the atomic orders and Mn-O bond lengths changed greatly compared with bulk MnO (Figure S18), indicating the instability of them. Therefore, the monomer Mn_1O_1 supported on Rh slab was chosen to represent the model Rh-MnO_x structure in subsequent calculations.

Boron oxide adsorption

To investigate the effect of boron oxide on the catalytic performance of RhMn/SiO_2, we first explored the adsorption strength of boron oxide on varied Mn_1O_1 supported Rh facets. The (100), (111), (221) and (322) were considered as main adsorption facets according to previous Wulff construction. Figure S19 shows the most stable B_2O_3 adsorbed sites on (100), (111), (221) and (322) facets. We defined the adsorption energy (ΔE) as following:

$$\Delta E = E_{\mathrm{tot}} - E_{\mathrm{slab}} - E_{\mathrm{adsorbate}}$$

where E_{tot} is the total DFT-calculated energy of the Mn_1O_1 supported Rh slab with the adsorbed species, E_{slab} is the DFT-calculated energy of the Mn_1O_1 supported Rh slab, and $E_{\mathrm{adsorbate}}$ is the DFT-calculated energy of the adsorbed species.

The adsorption energies relative to (322) facet (ΔE-ΔE_{322}) was listed in Table S9. In the same way, the adsorption energies of CO were also calculated and listed in Table S9.

Energy barriers of syngas conversion intermediate steps

In the syngas conversion process,[17,18] the first intermediate step is *CO + *H → *CHO or *CO → *C + *O while the latter one has a much higher energy barrier[19,20] (Entry 1-2 in Table S10), resulting in *CO + *H → *CHO being the first step generally. The C-O cleavage of $*CH_xOH$ → $*CH_x$ + *OH generates $*CH_x$ that is a key intermediate for C_1 products and C_2-oxygenates. $*CH_xCO$ generated through the C-C coupling of $*CH_x$ + *CO → $*CH_xCO$ and $*CH_xCHO$ generated through the C-C coupling of $*CH_x$ + *CHO → $*CH_xCHO$ are generally considered as the main precursors for C_2-oxygenates.[21,22] For simplicity, *CO + *H → *CHO, $*CH_2OH$ → $*CH_2$ +*OH, $*CH_2$ + *CO → $*CH_2CO$ and $*CH_2$ + *CHO → $*CH_2CHO$ were chosen as four representative intermediate steps to investigate so as to demonstrate the origins of enhanced C_2-oxygenates selectivity and the reduced overall activity due to the deposition of boron oxide on the surface of Rh-Mn catalysts (Figures S20-S23).

Catalytic reaction

The conversion of syngas into ethanol was performed in a fixed-bed stainless steel reactor mounted vertically (length = 600 mm; inside diameter = 8.0 mm). For each reaction run, 0.5 g of catalyst particles (20-40 mesh) were mixed with 1.5 g of quartz sand (20-40 mesh) placed in the middle of the reactor. Prior to reaction tests, the catalyst was reduced in pure H_2 at 400 °C for 2 h (flow rate at 50 mL/min). After the cooling reactor to 320 °C, the feed gas (H_2/CO ratio at 2/1, molar, flow rate of 28 mL/min) was instructed into the reactor and started at a pressure of 3.0 MPa. The gas phase products were analyzed by two gas chromatographs. The one is an online gas chromatograph (Fu Li-9790 II) equipped with a thermal conductivity detector (TCD) and a TDX-01 column (3 m × 3 mm). The other one is an online gas chromatograph (Fu Li-9790PLUS) equipped with a flame ionization detector (FID) and two columns (FFAP, 30 m × 0.32 mm × 0.25 μm and PLOT-Al_2O_3, 50 m × 0.53 mm × 0.25 μm). Argon with a concentration of 4 % in the reactant mixture was spent as an internal standard for calculating CO conversion in TCD. CH_4 was spent as an internal standard for calculating the product selectivities detected by online FID. The liquid phase products collected by cold trap were analyzed by an offline chromatograph (Fu Li-9790PLUS) equipped with a capillary column FFAP (30 m × 0.32 mm × 0.25 μm) and an FID. The conversions and product selectivities were based on the amount of carbon atoms.

CO conversion was calculated from eq (1) in the following:

$$\text{CO conversion} = 1 - \frac{A(\text{CO})_{out}/A(\text{Ar})_{out}}{A(\text{CO})_{in}/A(\text{Ar})_{in}} \times 100\%$$

Where $A(\text{CO})_{out}$ and $A(\text{Ar})_{out}$ represent the chromatographic peak areas of CO and Ar in the off-gas, and the $A(\text{CO})_{in}$ and $A(\text{Ar})_{in}$ represent the chromatographic peak areas of CO and Ar in the feed gas.

The product selectivity was calculated from eq (2) in the following:

$$Sel_{.Ci} = \frac{C_i \times i}{\sum(C_i \times i)} \times 100\%$$

Where C_i is the number of moles generated per hour of C_i product containing i carbon atoms. The selectivity of C-oxygenates (abbr. C-oxy) is defined as the ratio of total amount of oxygenates to all products, the selectivity of C_{2+}-oxygenates (abbr. C_{2+}-oxy) is defined as the ratio of total oxygenate products except for methanol to all products, and the distribution of C_2-oxygenates (abbr. C_2-oxy) describes the proportion of oxygenates (acetaldehyde, methyl formate, methyl acetate, ethyl acetate, and ethanol) in all the products.

Supplemental Tables and Figures

Table S1. Data characterizing the performances of various catalysts in the conversion of syngas to ethanol[a]

Entry	Catalyst	CO Conv. (%)	Selectivity (%)						
			CH_4	CO_2	CH_3OH	EtOH	C_2-oxy[b]	C-oxy[c]	CH(C>1)
1	Rh/SiO_2	1.7	62.4	11.2	5.2	8.4	12.3	17.9	8.5
2	$RhMn/SiO_2$	15.6	54.4	5.9	2.9	16.7	30.5	33.7	6.1
3	$RhMnB_{2.1}/SiO_2$	14.9	46.4	3.4	3.7	26.9	42.2	46.3	3.8
4	$RhMnB_{3.9}/SiO_2$	12.6	31.1	2.0	5.7	38.5	57.6	63.9	3.0
5	$RhMnB_{9.4}/SiO_2$	12.0	39.9	2.3	5.3	32.4	48.1	53.9	3.9
6	$RhMnNaB_{3.8}/SiO_2$	8.3	26.1	4.8	5.9	39.0	59.4	66.0	3.0

[a] Reaction conditions: 0.5 g of catalyst, H_2/CO at 2, 3360 mL h^{-1} g^{-1}, 3 MPa, 320 °C.

[b] Selectivity of C_2-oxygenates (acetaldehyde, methyl formate, methyl acetate, ethyl acetate, and ethanol) in all the products.

[c] Total oxygenate products.

Table S2. Metal loadings and average Rh sizes of various catalysts.

Catalyst	Rh loading (wt%)[a]	Mn loading (wt%)[a]	B loading (wt%)[a]	Mole ratio of B/Rh	Average Rh diameters (nm)[b]
$RhMn/SiO_2$	0.74	0.40	-[c]	0	2.3
$RhMnB_{2.1}/SiO_2$	0.82	0.45	0.18	2.1	1.9
$RhMnB_{3.9}/SiO_2$	0.81	0.43	0.33	3.9	1.7
$RhMnB_{9.4}/SiO_2$	0.72	0.45	0.71	9.4	1.6
$RhMnNaB_{3.8}/SiO_2$	0.76	0.38	0.30	3.8	1.6
used-$RhMn/SiO_2$	0.76	0.41	-[c]	0	3.5
used-$RhMnB_{3.9}/SiO_2$	0.77	0.43	0.30	3.7	2.3

[a] By ICP analysis. Each sample was analysed 3 times to obtain the average metal loadings with the error bounds at ± 0.05%.

[b] Rh nanoparticle size distributions were obtained by counting more than 200 nanoparticles for each sample. Average Rh diameters were calculated *via* Gaussian distribution method in the software ORIGIN 2018.

[c] Undetectable B.

Table S3. Data characterizing catalytic performances of the catalysts under different reaction temperatures[a]

Catalyst	CO Conv. (%)	Selectivity (%)						
		CH_4	CO_2	CH_3OH	EtOH	C_{2+}-oxy[b]	C-oxy[c]	CH(C>1)
$RhMn/SiO_2$[d]	9.6	47.4	0.5	4.4	29.4	36.2	40.6	11.6
$RhMnB_{3.9}/SiO_2$[d]	9.5	41.4	0.1	7.5	32.4	45.4	52.9	5.6
$RhMn/SiO_2$[e]	5.3	38.1	0.0	8.6	33.0	45.8	54.4	7.6
$RhMnB_{3.9}/SiO_2$[e]	5.2	36.8	0.0	10.2	34.8	46.3	56.5	6.8

[a] Reaction conditions: 0.5 g of catalyst, H_2/CO at 2, 3240 mL h^{-1} g^{-1}, 3 MPa, 320 °C.

[b] Total oxygenate products except for methanol.

[c] Total oxygenate products.

[d] Reaction temperature: 300 °C.

[e] Reaction temperature: 280 °C.

Table S4. Data characterizing the performances of $RhMnB_{3.9}/SiO_2$ catalysts with different ratios of H_2/CO in the conversion of syngas to ethanol [a]

Entry	Ratio of H_2/CO	CO Conv. (%)	Selectivity (%)						
			CH_4	CO_2	CH_3OH	EtOH	C_2-oxy[b]	C-oxy[c]	CH(C>1)
1	1	10.7	17.2	1.7	7.7	29.2	56.7	65.8	15.3
2	2	12.6	31.1	2.0	5.7	38.5	57.6	63.9	3.1
3	3	35.4	49.5	6.3	8.5	20.7	28.5	37.3	6.9

[a] Reaction conditions: 0.5 g of catalyst, 28 mL/min, 3 MPa, 320 °C.

[b] Selectivity of C_2-oxygenates (acetaldehyde, methyl formate, methyl acetate, ethyl acetate, and ethanol) in all the products.

[c] Total oxygenate products.

Table S5. Data showing the performances of various catalysts reported in literature.

Catalyst	H_2/CO	Temp. (°C)	Pressure (MPa)	WHSV mL $g_{cat}^{-1}h^{-1}$	CO conv. (%)	Selectivity (%)				Ref
						MeOH	C_{2+}-oxy[a]	CH[b]	CO_2	
Rh@S-1	2	250	5	6000	4.1	9.0	27.0	64.0	0	23
0.19Na-Rh@S-1	2	300	5	6000	3.8	60.0	24.0	16.0	0	23
7Rh/NFe_2O_3	0.5	240	0.1	1100	2.0	2.7	32.5	64.9	-	24
RM-*in*-CNT	2	320	3	12000 h^{-1}	18.8	2.2	40.1	56.6	1.1	25
Rh-0.15Fe-0.10Mn	1	250	4	-	1.2	5.6	39.6	39.8	15.9	26
Rh/Al_2O_3	2	260	2	3600	11.4	8.9	18.1	62.1	9.9	27
Rh/ZrO_2	2	275	2.4	2240	18.2	1.9	38.6	58.0	2.5	28
Rh/TiO_2	2	300	3	2400 h^{-1}	5.5	5.2	15.8	73.1	5.9	29
Rh-Fe/CeO_2	2	290	3	5000 h^{-1}	5.9	14.8	35.3	33.3	16.5	30
Rh/Mn/SiO_2	2	277	8.3	7500	20.8	0.5	51.6	47.9	0	31
Rh/Fe/Al_2O_3	2	280	5	12500 h^{-1}	5.2	24.0	29.2	40.2	1.8	32
RhFe@SiO_2	2	270	2.5	4800	8.4	16.4	38.2	45.3	0	33
$RhMnB_{3.9}/SiO_2$	2	320	3	3360	12.6	5.7	58.2	34.2	2.0	TW
$RhMnNaB_{3.8}/SiO_2$	2	320	3	3360	8.3	5.9	60.1	29.1	4.8	TW

[a] Total oxygenate products except for methanol.

[b] Total hydrocarbon products.

Table S6. Relative surface atomic ratios of different catalysts.

Catalyst	$Rh^{\delta+}$ (Atomic %)	Rh^0 (Atomic %)	$Rh^{\delta+}/Rh^0$
$RhMn/SiO_2$	37.9	62.1	0.61
$RhMnB_{3.9}/SiO_2$	40.2	59.8	0.67
Used-$RhMn/SiO_2$	20.2	79.8	0.25
Used-$RhMnB_{3.9}/SiO_2$	26.8	73.2	0.37

Table S7. EXAFS fit parameters of $RhMn/SiO_2$ and $RhMnB_{3.9}/SiO_2$ before and after reaction.[a]

Catalyst	Rh-O		Rh-Rh		σ^2 (Å)	ΔE_0 (eV)
	R (Å)	*CN*	*R (Å)*	*CN*		
$RhMn/SiO_2$	2.03±0.06	2.99±0.22	2.68±0.02	0.94±0.36	0.003(O)<br>0.006(Rh)	3.10±2.30
used-$RhMn/SiO_2$	1.83±0.01<br>2.01±0.05	0.42±0.33<br>2.46±0.36	2.68±0.03	2.51±0.28	0.003(O)<br>0.006(Rh)	0.32±1.65
$RhMnB_{3.9}/SiO_2$	2.03±0.07	3.39±0.15	2.66±0.06	0.72±0.23	0.003(O)<br>0.006(Rh)	3.53±1.31
used-$RhMnB_{3.9}/SiO_2$	1.83±0.01<br>2.02±0.05	0.91±0.29<br>2.85±0.33	2.66±0.04	2.69±0.36	0.003(O)<br>0.006(Rh)	1.13±1.87

[a] CN is the coordination number; R is interatomic distance; σ^2 is Debye-Waller factor; ΔE_0 is edge-energy shift. S_0^2 is the amplitude reduction factor (S_0^2=0.81), Data ranges1.0≤ R ≤3.0Å, 3.0≤ k ≤10.0 Å. Estimated EXAFS error bounds: N ± 20%; R ± 1%; σ^2 ± 20%; ΔE_0 ± 20%. XAS data were processed and analyzed using the Demeter software package.[34]

Table S8. The area percentage of different crystal facets of Rh Wulff construction.

Facet	Percentage (%)	Surface energy (eV/Å^2)
(111)	45.8	0.147017748
(221)	16.6	0.162352573
(322)	13.9	0.159720087
(100)	11.9	0.171877914
(311)	8.3	0.173937757
(310)	2.5	0.180038290
(110)	0.9	0.171808288

Table S9. The adsorption energy (eV) of B_2O_3 and CO on four representative Rh facets.

Facet	ΔE_{hkl}-ΔE_{322}/eV		CN	GCN
	B_2O_3	CO		
(111)	0.36	0.15	9	7.50
(100)	0.20	0.14	8	6.67
(221)	0.15	0.02	7(E), 9(T)	5.50(E), 7.17(T), 7.83(T), 9.50 (T)
(322)	0.00	0.00	7(E), 9(T), 10(T)	5.50(E), 7.17(T), 7.50(T), 7.67(T), 8.75(T)

Note: CN is the coordination number of Rh and GCN[35] is the generalized coordination number. For CN and GCN, E stands for the edge site while T stands for the terrace site on the corresponding stepped facets.

Table S10. The Gibbs free energy activation barriers (ΔG_a) of key intermediate steps on Mn_1O_1 supported Rh (111) and (221) facets at 595 K.

Entry	Steps	ΔG_a /eV	
		(111)	(221)
1	$*CO \rightarrow *C + *O$	2.97	2.70
2	$*CO + *H \rightarrow *CHO$	1.21	1.03
3	$*CHO + *H \rightarrow *CH_2O$	0.66	0.61
4	$*CHO + *H \rightarrow *CHOH$	0.76	0.82
5	$*CH_2O + *H \rightarrow *CH_3O$	0.56	0.59
6	$*CH_2O + *H \rightarrow *CH_2OH$	0.76	0.77
7	$*CH_2OH \rightarrow *CH_2 + *OH$	0.76	0.71
8	$*CH_3O \rightarrow *CH_3 + *O$	1.57	1.40
9	$*CH_2 + *H \rightarrow *CH_3$	0.49	0.40
10	$*CH_2 + *CO \rightarrow *CH_2CO$	1.21	1.39
11	$*CH_2 + *CHO \rightarrow *CH_2CHO$	0.63	0.69

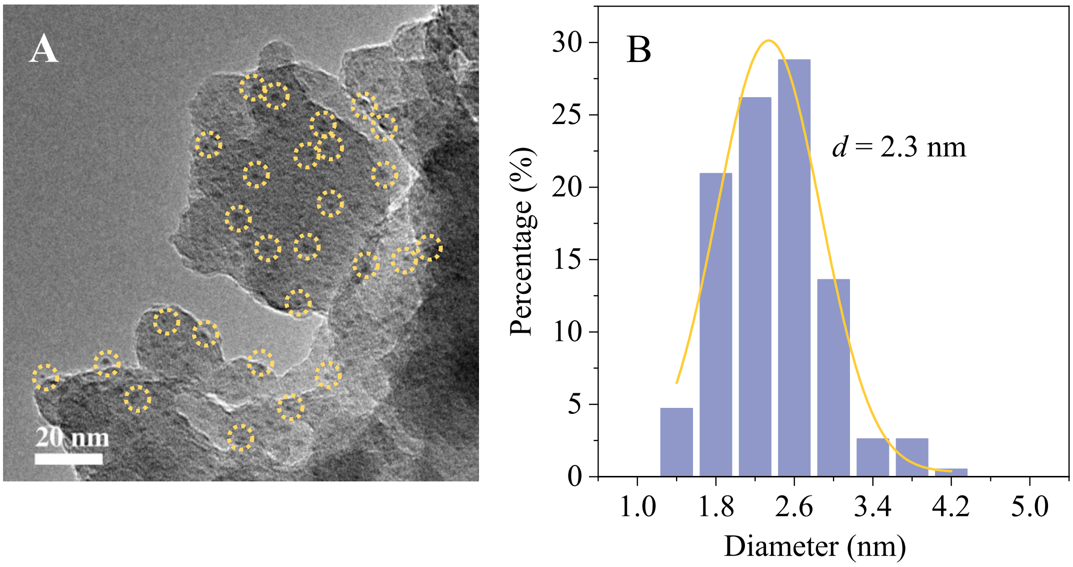

Figure S1. (A) TEM image and (B) RhMn nanoparticle size distribution of the RhMn/SiO_2 catalyst. The Rh NPs in Figure S1A are highlighted by the yellow circles. Rh nanoparticle size distributions were obtained by counting more than 200 nanoparticles for each sample. Average Rh diameters were calculated *via* Gaussian distribution method in the software ORIGIN 2018.

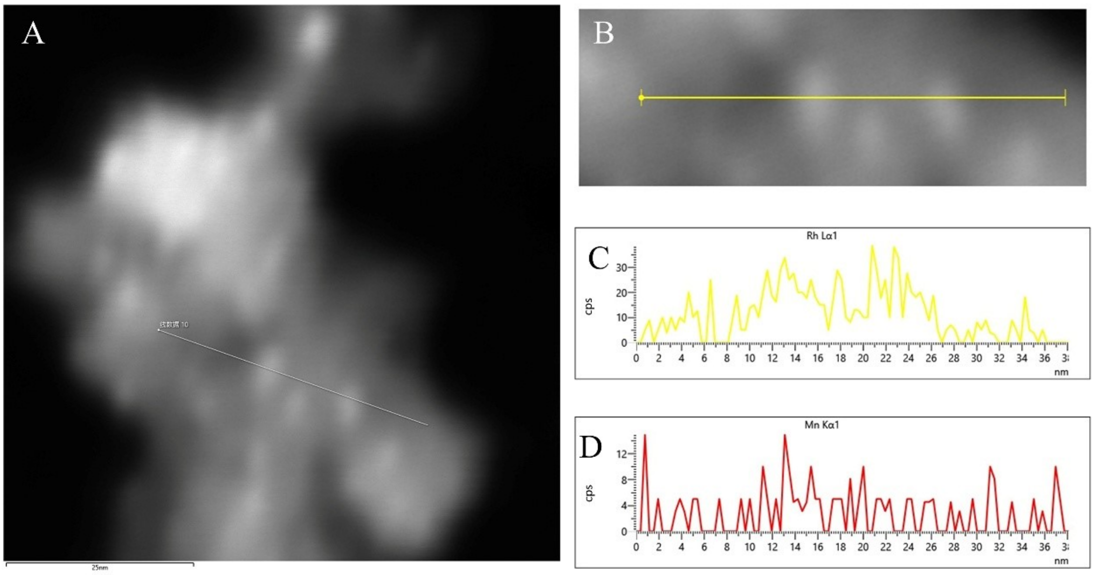

Figure S2. (A and B) STEM images and (C and D) linear-scan EDS spectra of the RhMn/SiO_2 catalyst.

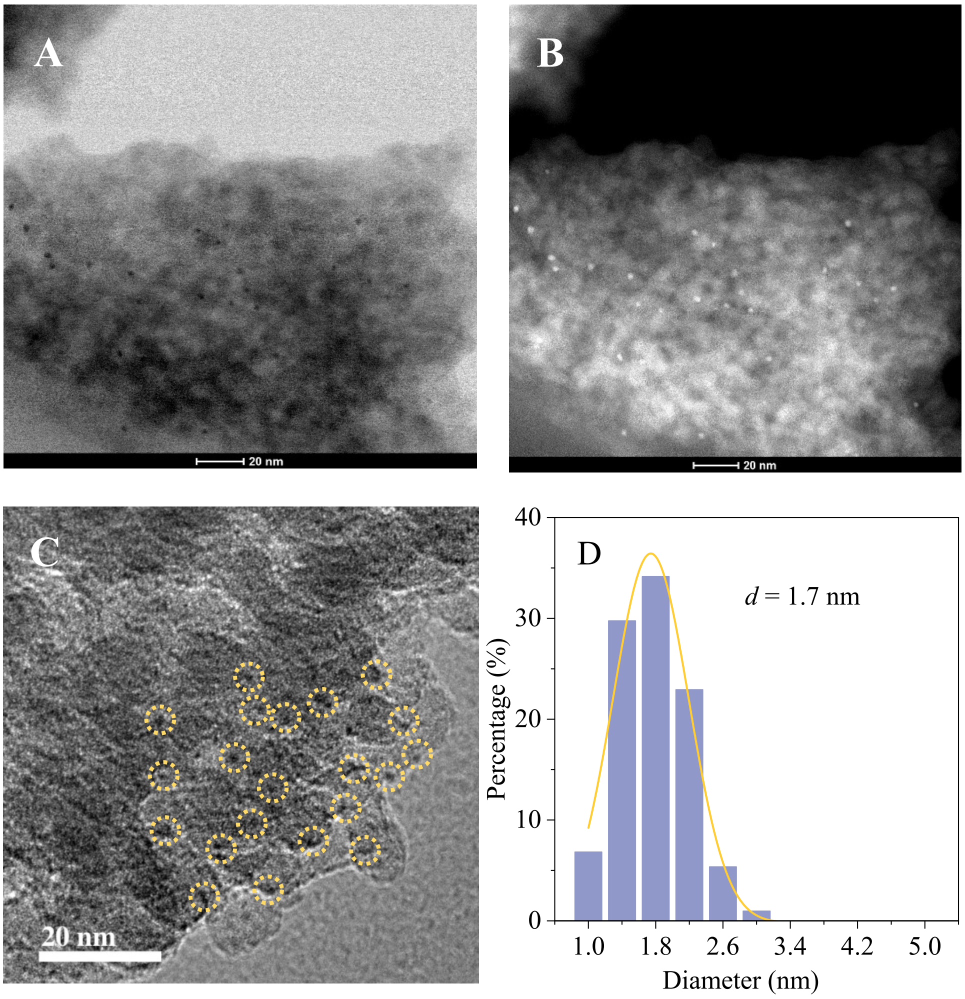

Figure S3. (A) BF-STEM image, (B) HAADF-STEM image, (C) TEM image, and (D) RhMn nanoparticle size distribution of the $RhMnB_{3.9}/SiO_2$ catalyst. The Rh NPs in Figure S3C are highlighted by the yellow circles.

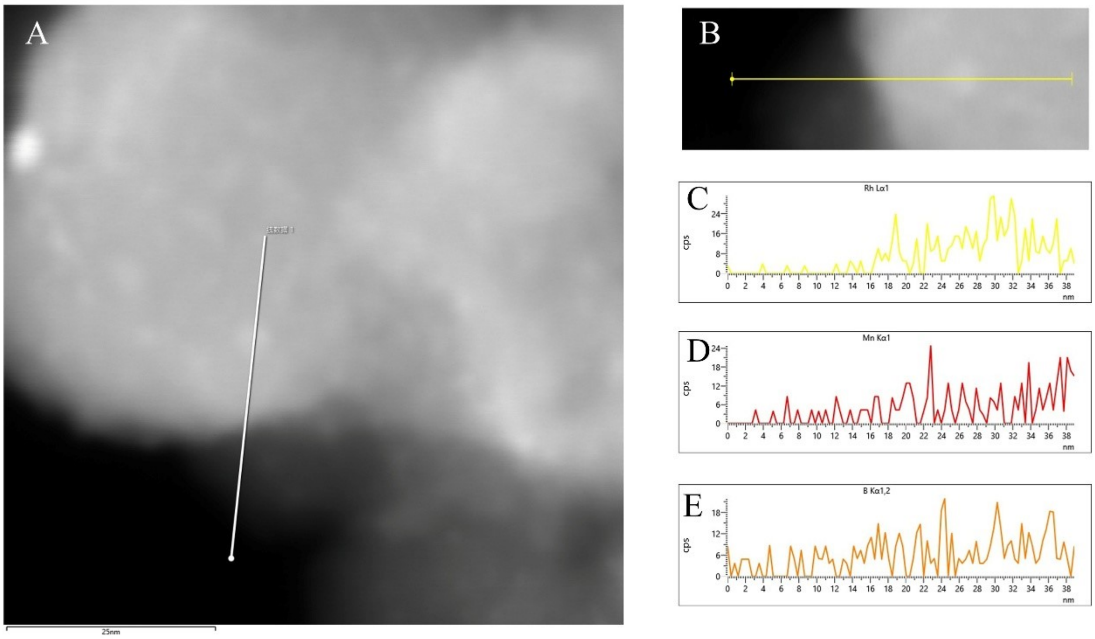

Figure S4. (A and B) STEM images and (C-E) linear-scan EDS spectra of the $RhMnB_{3.9}/SiO_2$ catalyst.

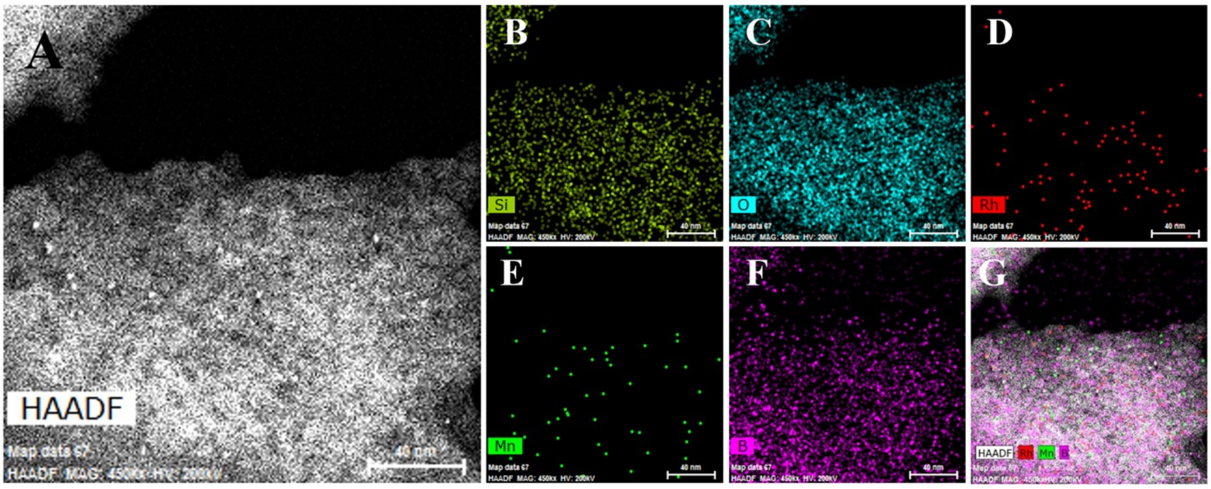

Figure S5. (A) HAADF-STEM image and EDS elemental maps of (B) Si, (C) O, (D) Rh, (E) Mn, (F) B, and (G) their overlap signals of the $RhMnB_{3.9}/SiO_2$ catalyst.

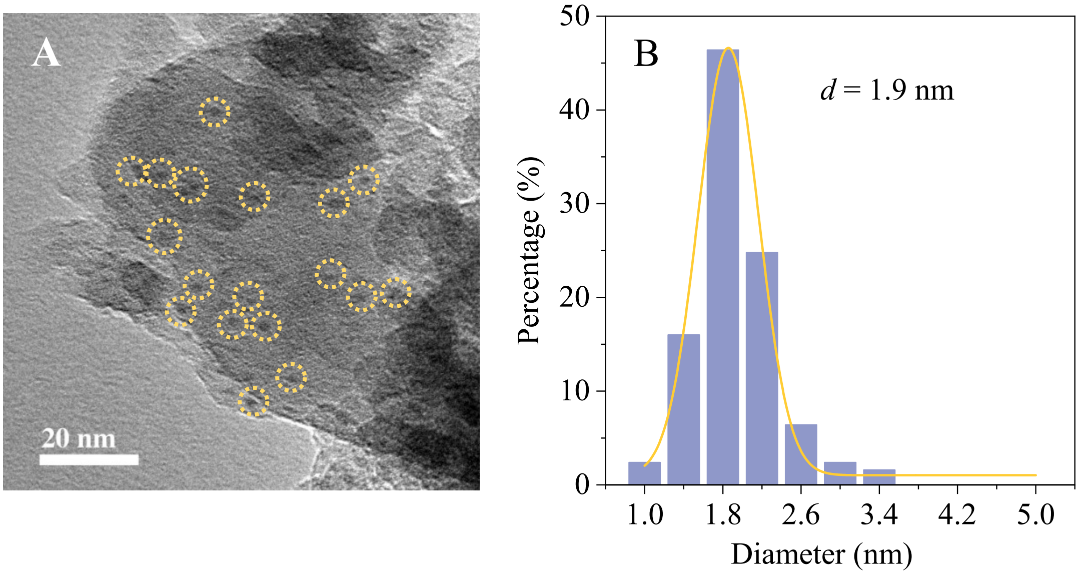

Figure S6. (A) TEM image and (B) RhMn nanoparticle size distribution of the $RhMnB_{2.1}/SiO_2$ catalyst. The Rh NPs in Figure S6A are highlighted by the yellow circles.

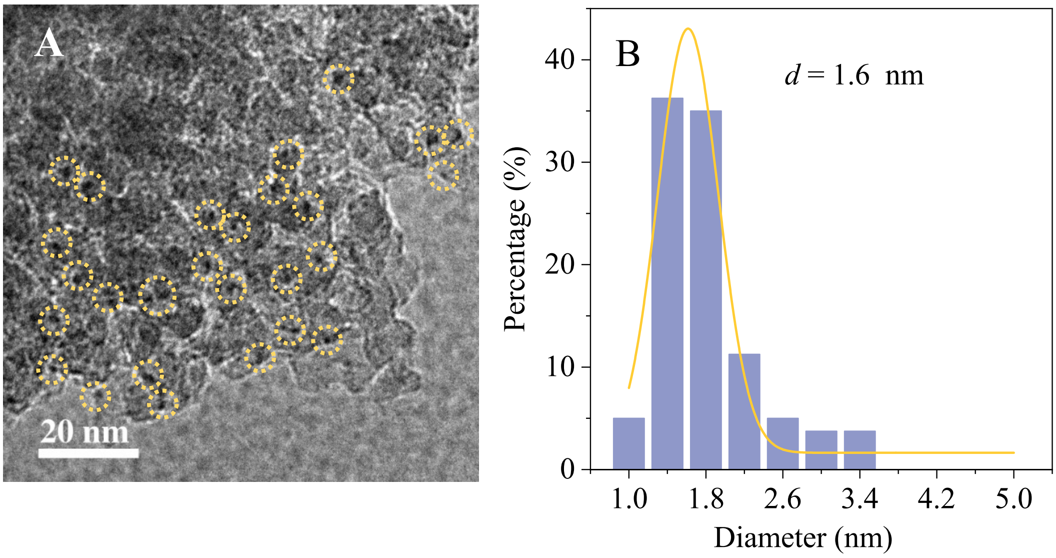

Figure S7. (A) TEM image and (B) RhMn nanoparticle size distribution of the $RhMnB_{9.4}/SiO_2$ catalyst. The Rh NPs in Figure S7A are highlighted by the yellow circles.

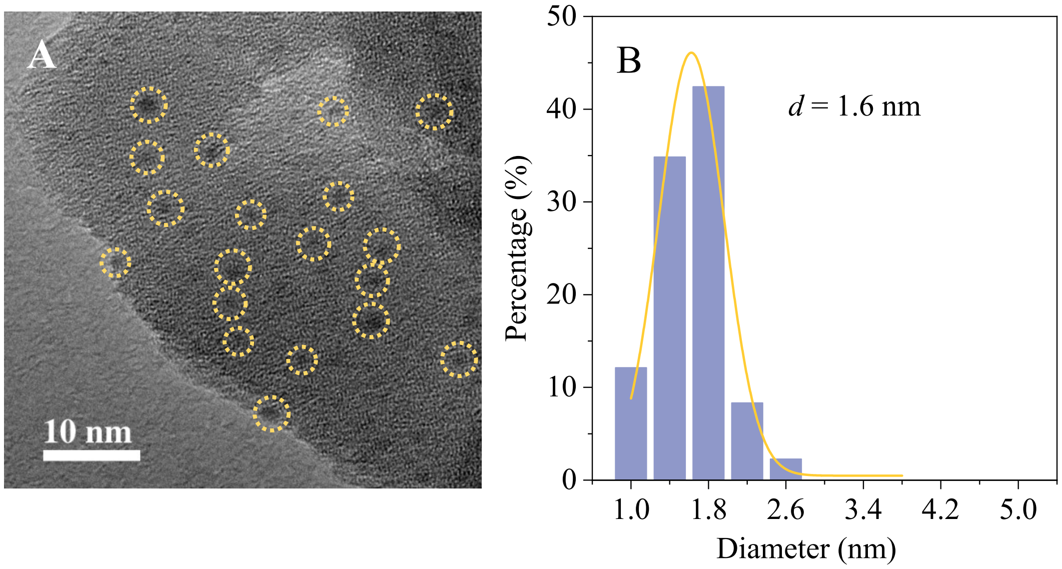

Figure S8. (A) TEM image and (B) RhMn nanoparticle size distribution of the $RhMnNaB_{3.8}/SiO_2$ catalyst. The Rh NPs in Figure S8A are highlighted by the yellow circles.

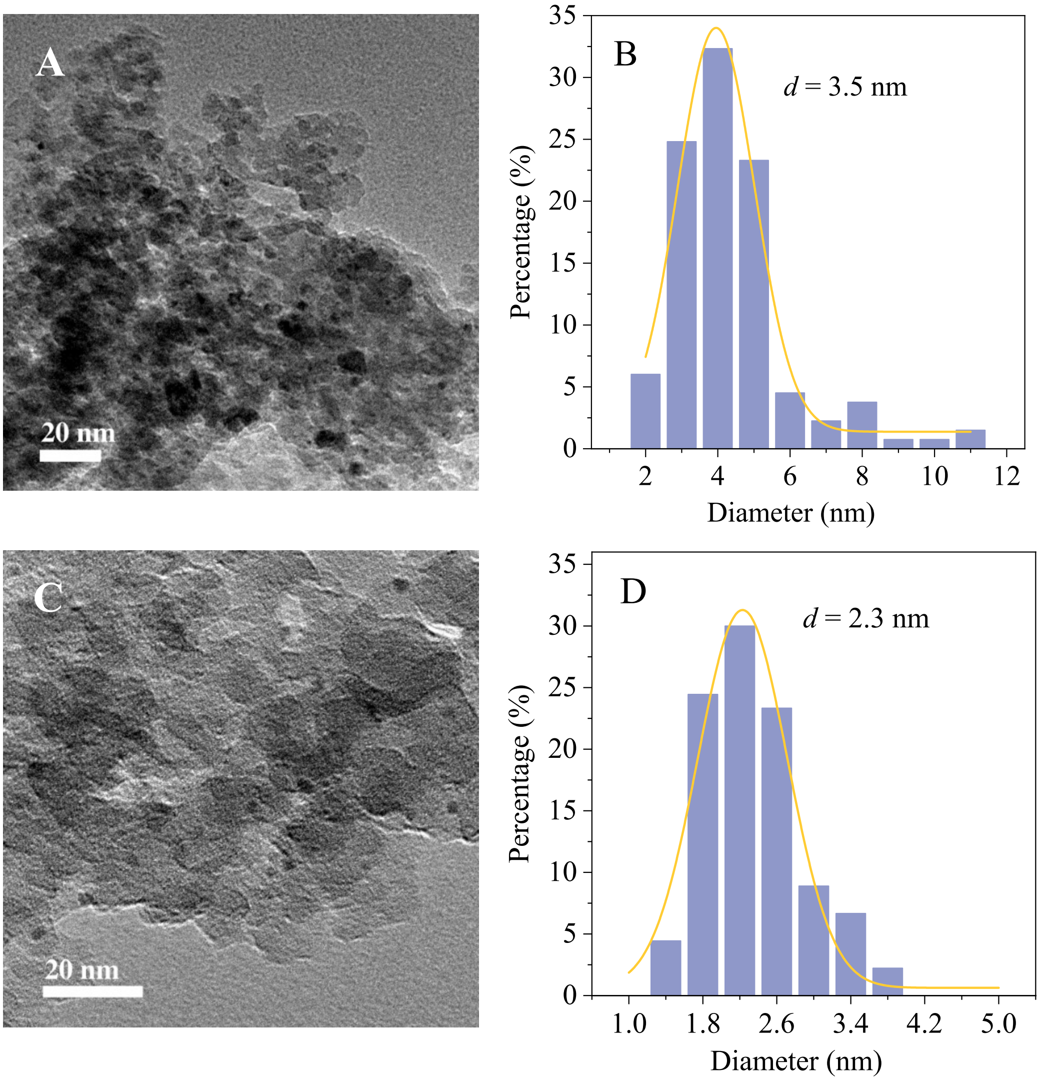

Figure S9. (A) TEM image and (B) RhMn nanoparticle size distribution of the used-RhMn/SiO_2 catalyst. (C) TEM image and (D) RhMn nanoparticle size distribution of the used-$RhMnB_{3.9}/SiO_2$ catalyst.

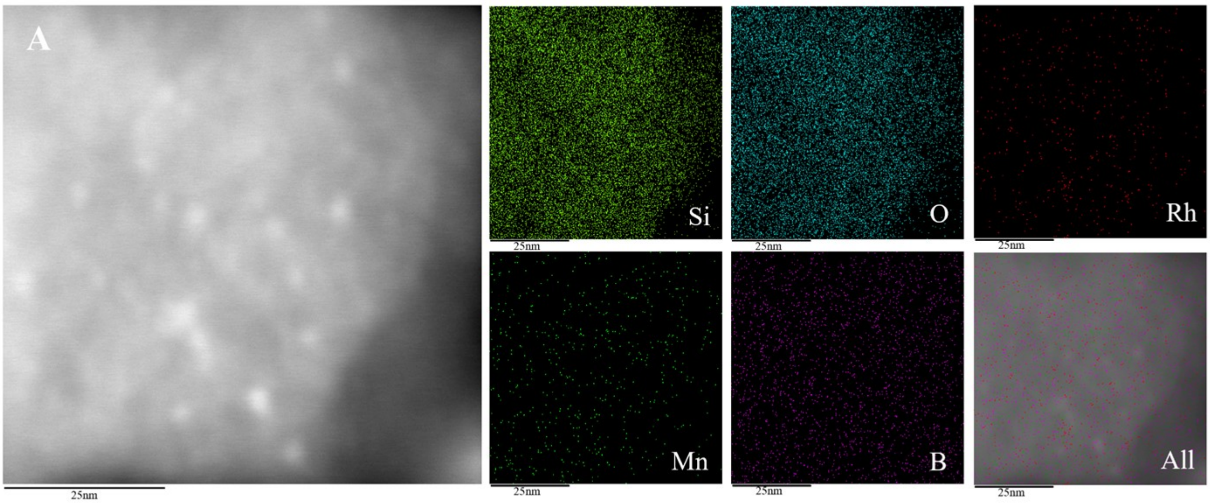

Figure S10. STEM image and EDS elemental maps of the used-$RhMnB_{3.9}/SiO_2$ catalyst.

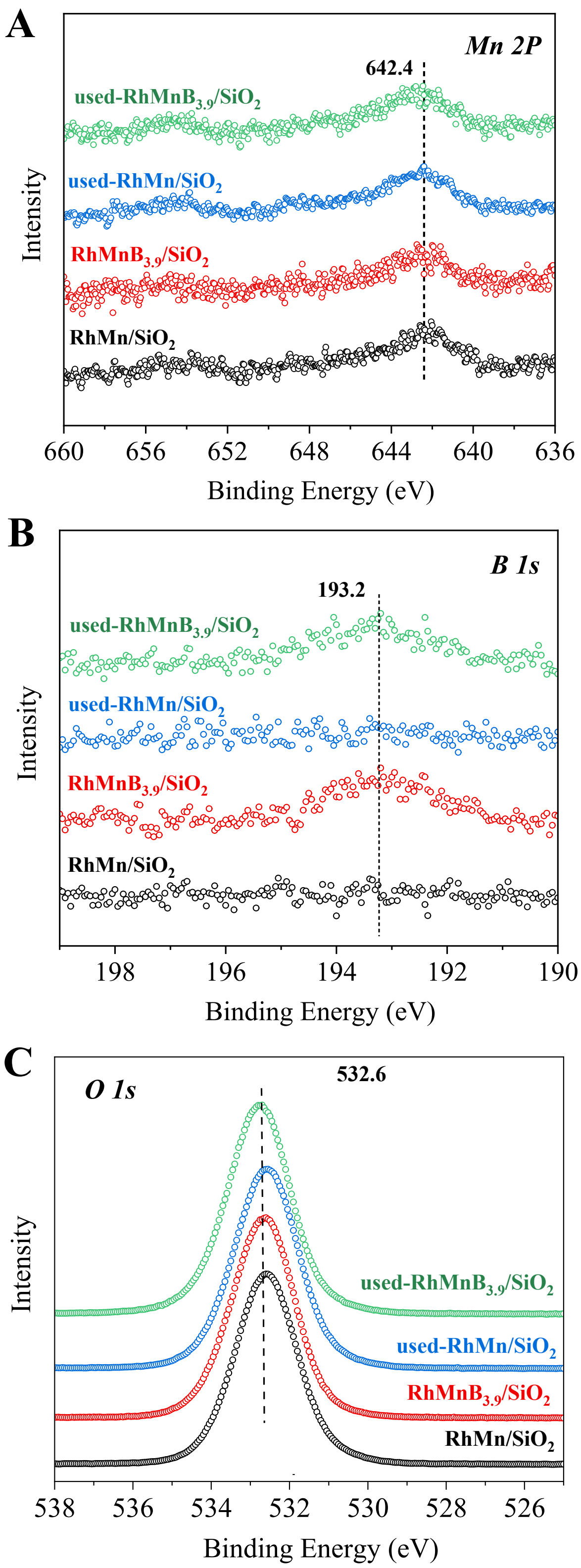

Figure S11. (A) Mn 2p, (B) B 1s, and (C) O 1s XPS spectra of the reduced and used $RhMn/SiO_2$ and $RhMnB_{3.9}/SiO_2$ catalysts.

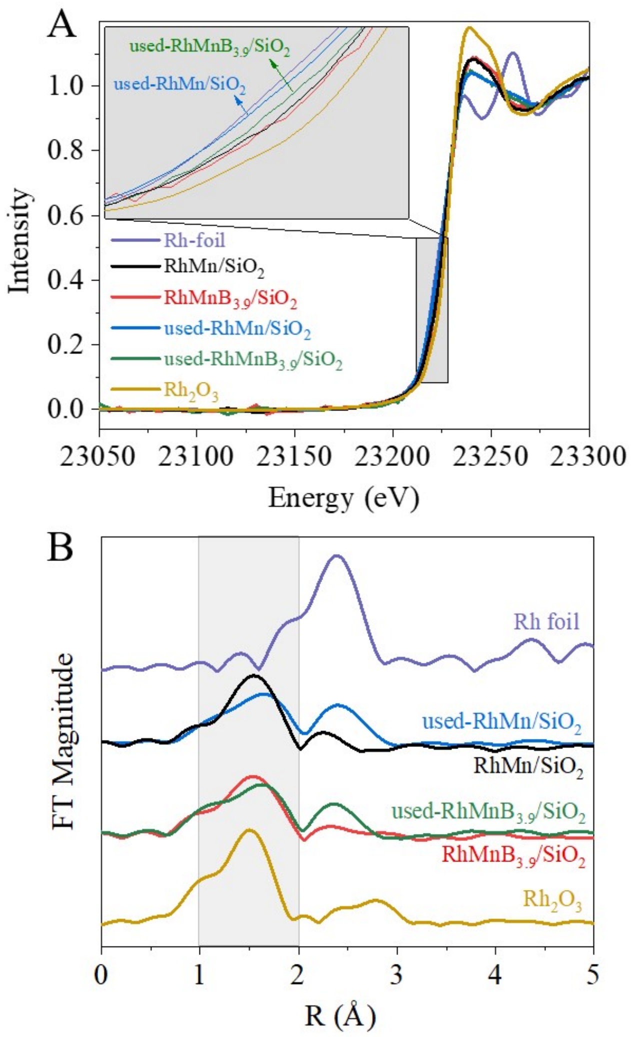

Figure S12. (A) XANES spectra at the Rh K-edge and (B) magnitude of the Fourier transform of the EXAFS spectra of the reduced and used RhMn/SiO_2 and $RhMnB_{3.9}/SiO_2$ catalysts. The inset in A showed an enlarged view of pre-edge energy.

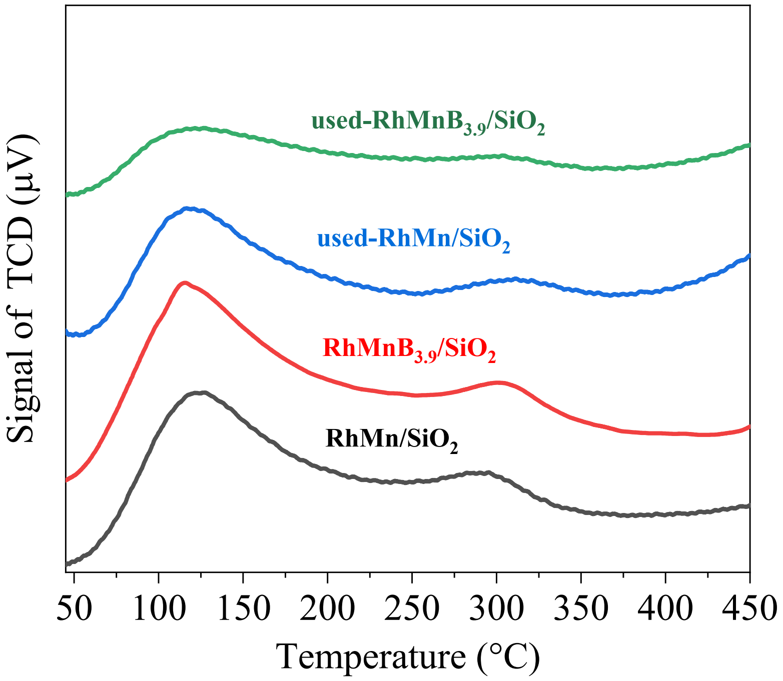

Figure S13. CO-TPD profiles of various catalysts.

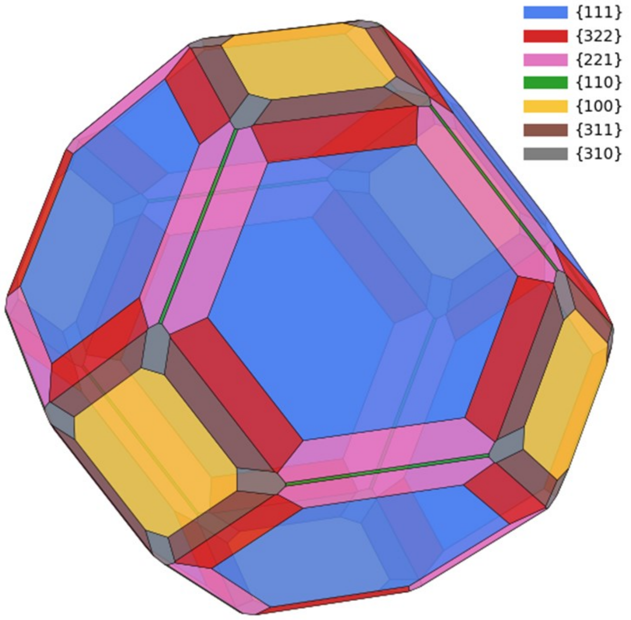

Figure S14. The equilibrium morphology of Rh Wulff construction.

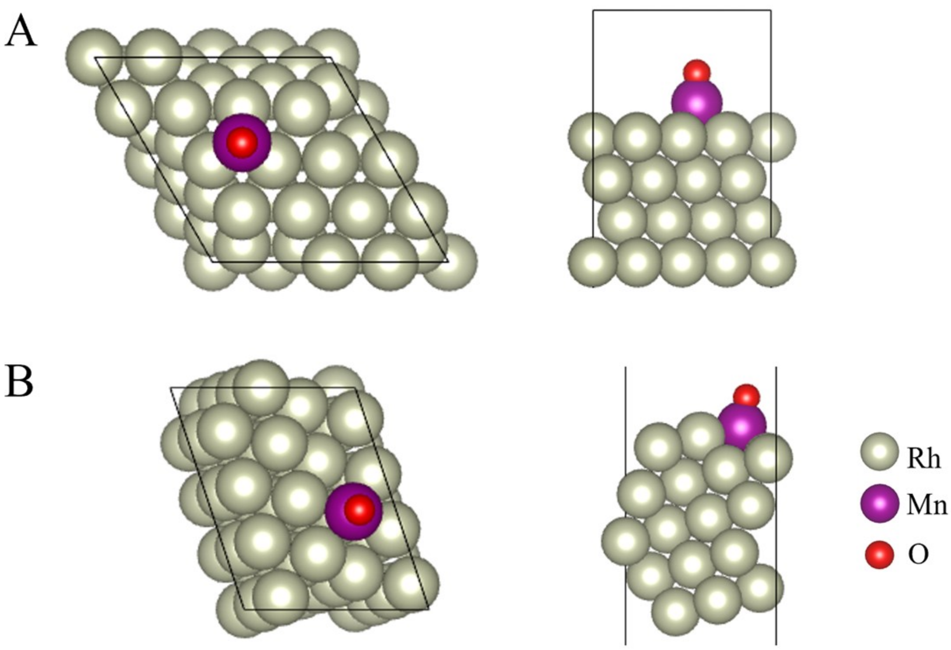

Figure S15. Mn_1O_1 supported on the (A) hcp site of Rh (111) facet; (B) fcc site of Rh (221) facet. (Left side is the top view and right side is the side view.)

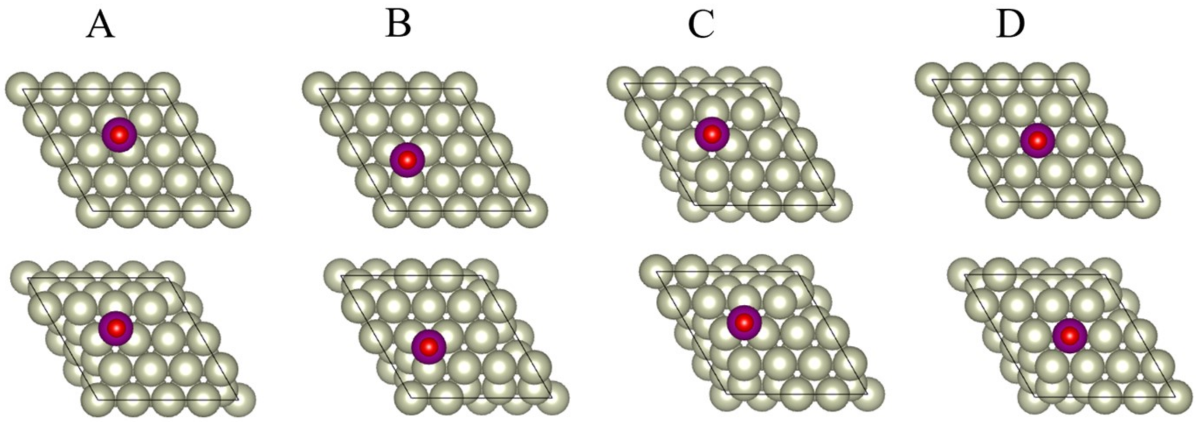

Figure S16. Mn_1O_1 supported sites on Rh (111) facet: (A) bridge; (B) fcc; (C) hcp; (D) top. (The first row is the structures before optimization, while the second row was the corresponding structures after optimization.)

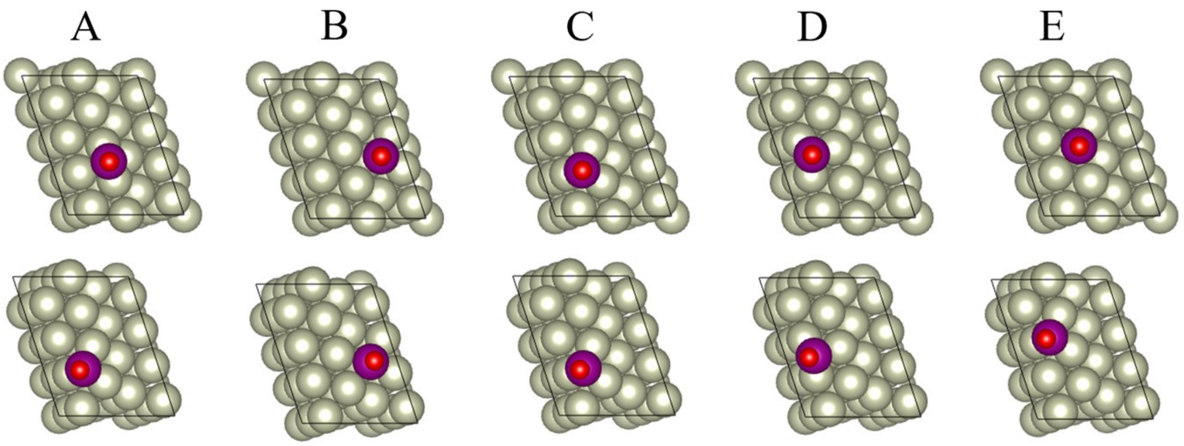

Figure S17. Mn_1O_1 supported sites on Rh (221) facet: (A) bridge; (B) fcc1; (C) fcc2; (D) hcp; (E) top. (The first row is the structures before optimization, while the second row is the corresponding structures after optimization.)

A

B

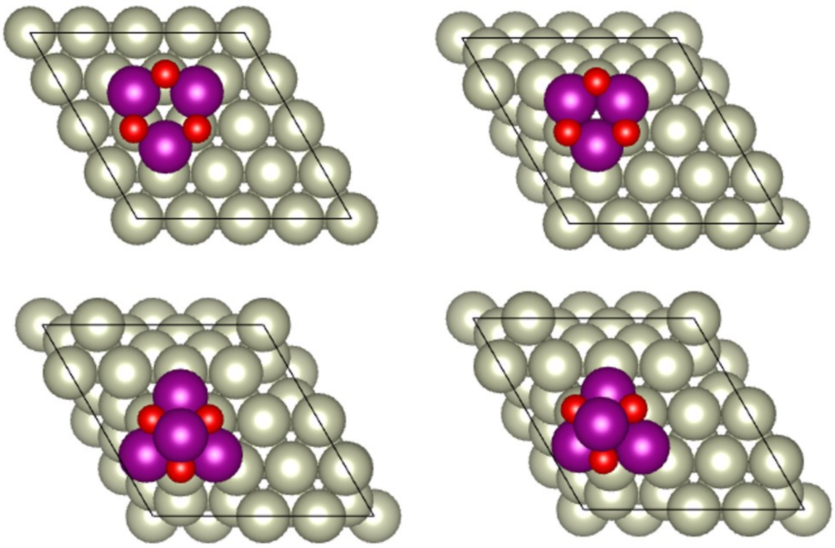

Figure S18. (A) Mn_3O_3 (B) Mn_4O_4 supported on Rh (111) facet. (The left column is the structures before optimization, while the right column is the corresponding structures after optimization.)

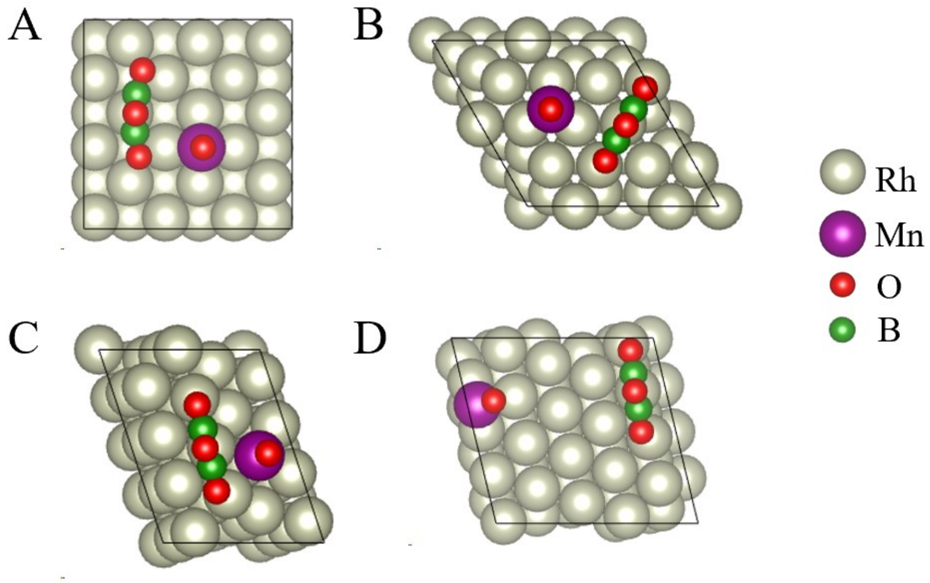

Figure S19. The most stable configurations of B_2O_3 adsorbed on Rh facets. (A), (B), (C) and (D) are corresponding to the Mn_1O_1 supported (100), (111), (221) and (322) facets of Rh, respectively.

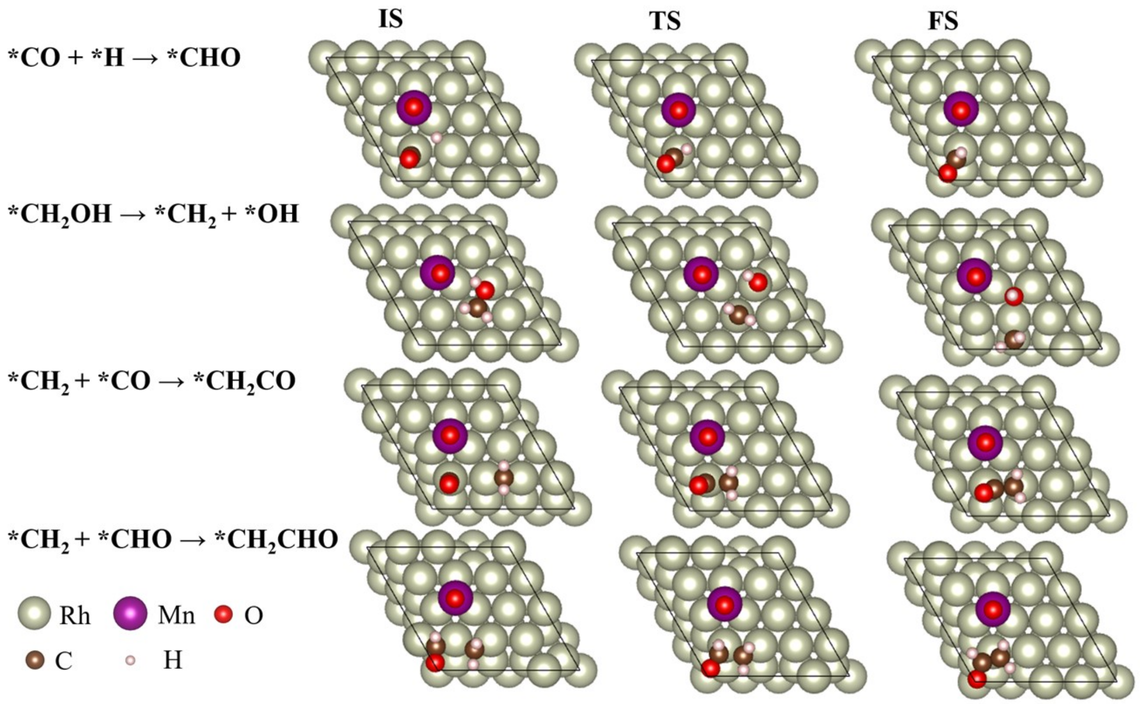

Figure S20. The configurations of IS, TS and FS of four intermediate steps on Rh (111) with Mn_1O_1 supported.

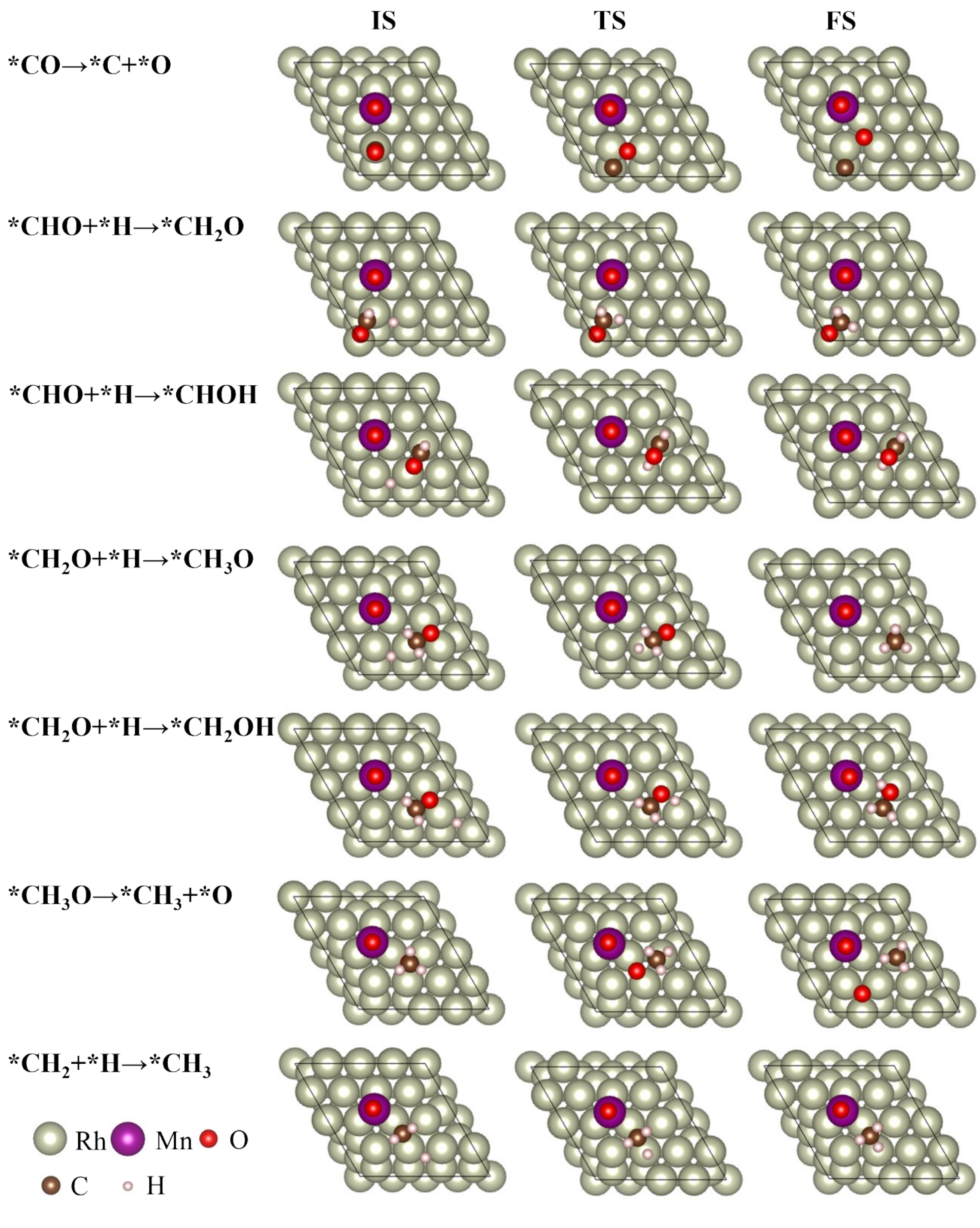

Figure S21. The configurations of IS, TS and FS for the remaining seven intermediate steps on Rh (111) with Mn_1O_1 supported.

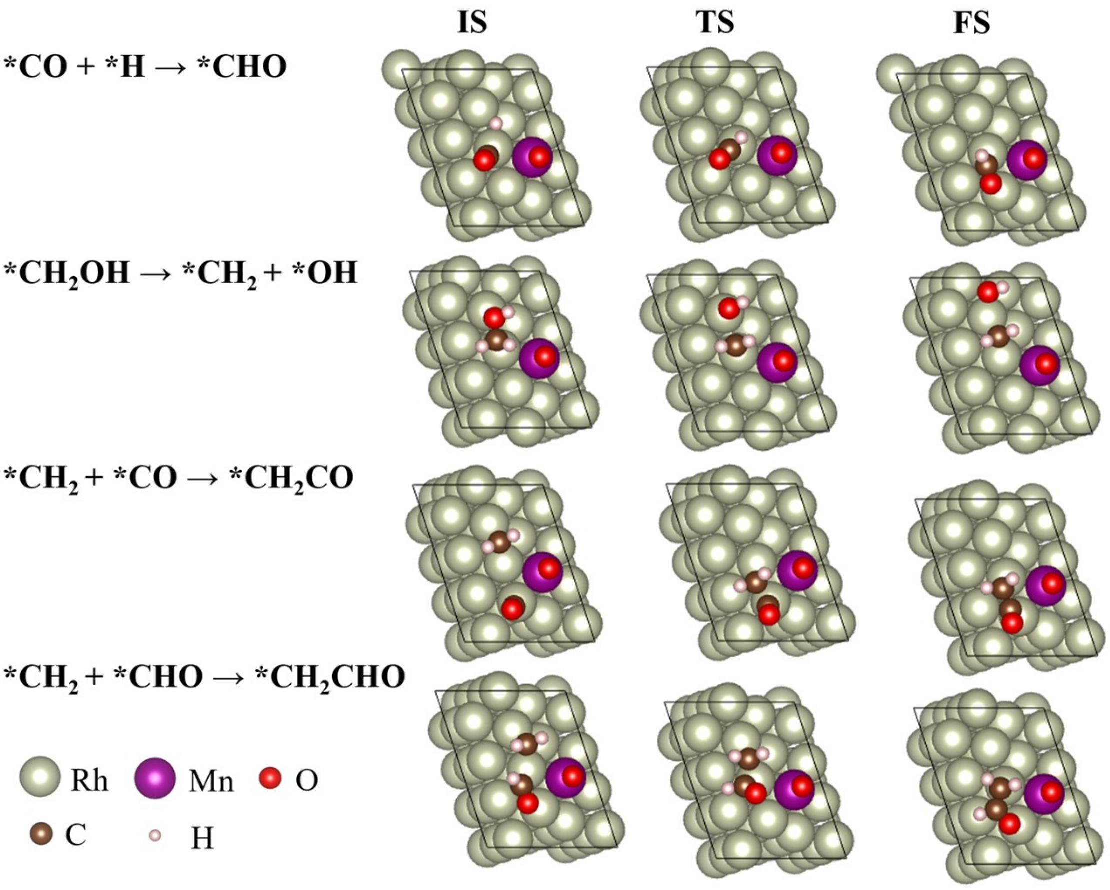

Figure S22. The configurations of IS, TS and FS of four intermediate steps on Rh (221) with Mn_1O_1 supported.

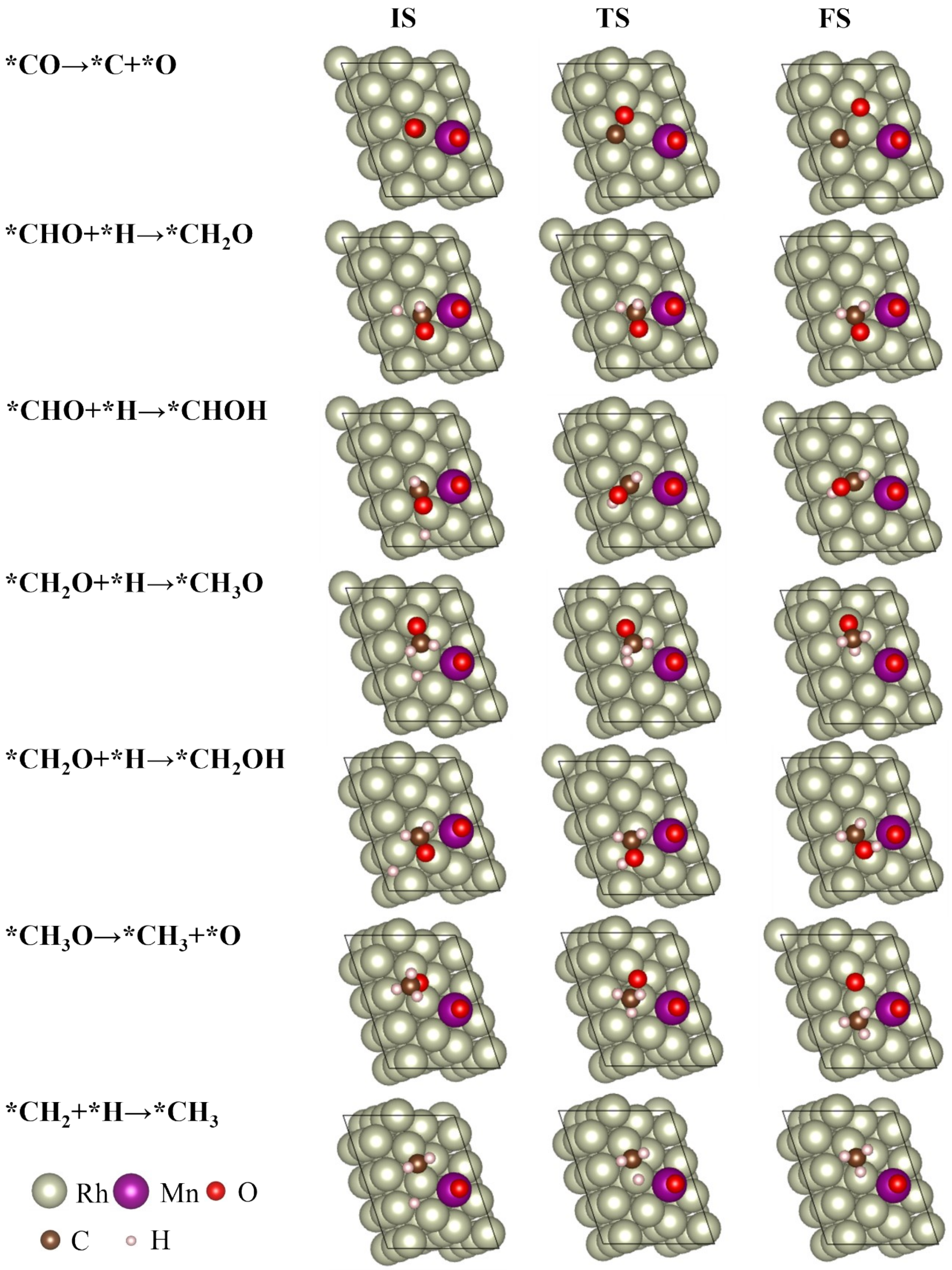

Figure S23. The configurations of IS, TS and FS for the remaining seven intermediate steps on Rh (221) with Mn_1O_1 supported.

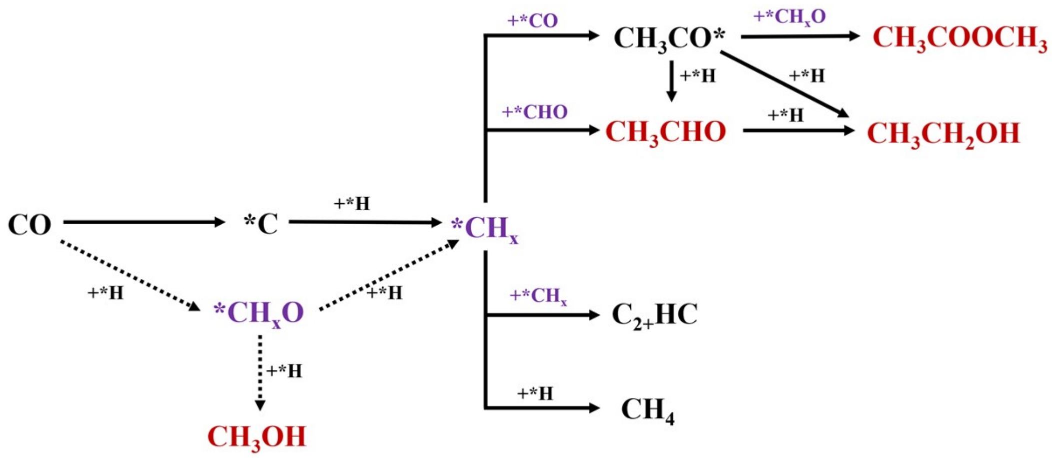

Figure S24. Schematic illustration of the proposed pathway ys of syngas conversion over the RhMn based catalysts.

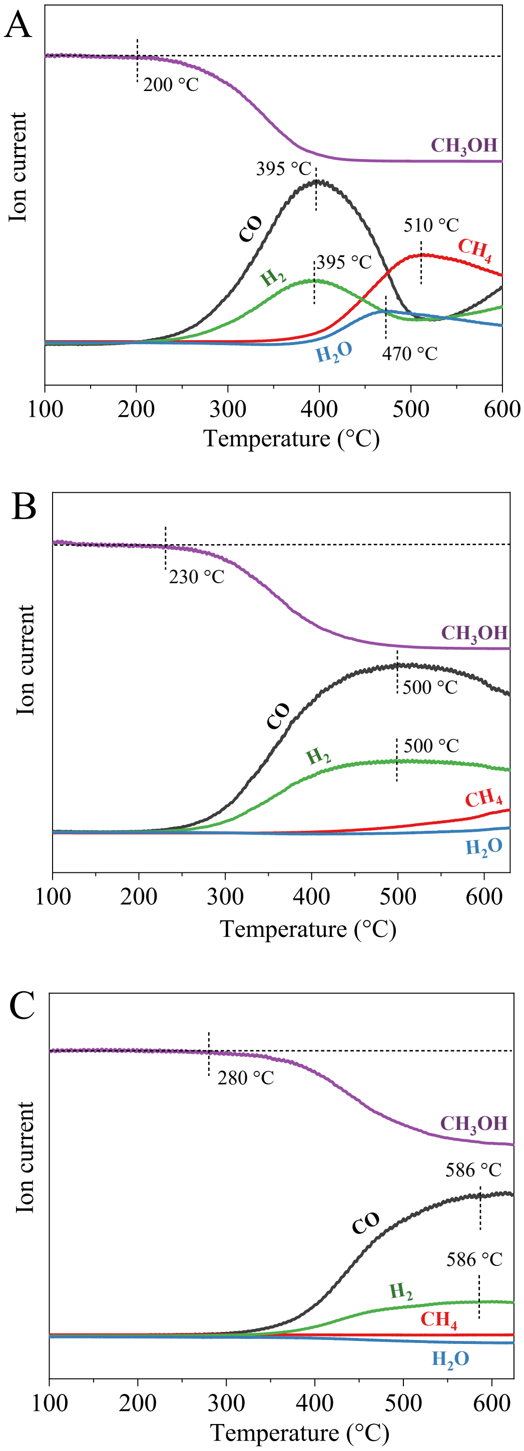

Figure S25. MeOH-TPSR profiles of the (A) $RhMn/SiO_2$, and (B) $RhMnB_{3.9}/SiO_2$ and (C) blank run.

Note: As a blank run, the signal of methanol was decreased at 280 °C, indicating that the methanol decomposition started to occur. The CO signal and H_2 signal centered at about 586 °C demonstrate the dehydrogenation of the $*CH_3O$ species indeed occurred at high temperature.

Reference

1 P. Hohenberg, W. Kohn. *Phys. Rev.* **1964**, *136*, B864-B871.

2 W. Kohn, L. J. Sham. *Phys. Rev.* **1965**, *140*, A1133-A1138.

3 P.E. Blöchl. *Phys. Rev. B* **1994**, 50, 17953-17979.

4 J. P. Perdew, K. Burke, M. Ernzerhof. *Phys. Rev. Lett.* **1996**, *77*, 3865-3868.

5 G. Kresse, J. Furthmüller. *Phys. Rev.* **1996**, *54*, 11169-11186.

6 J. D. Pack, H. J. Monkhorst. *Phys. Rev. B.* **1977**, *16*, 1748-1749.

7 M. Methfessel, A.T. Paxton. *Phys. Rev. B* **1989**, 40, 3616-3621.

8 G. Henkelman, B. P. Uberuaga, H. Jónsson. *J. Chem. Phys.* **2000**, *113*, 9901-9904.

9 G. Henkelman, H. Jónsson. *J. Chem. Phys.* **1999**, *111* (15), 7010-7022.

10 V. Wang, N. Xu, J.-C. Liu, G. Tang, W.-T. Geng. *Comput. Phys. Commun.* **2021**, *267,* 108033.

11 Momma, K., Izumi, F. *J. Appl. Crystallogr.* **2008**, 41, 653-658.

12 J. P. Perdew, A. Ruzsinszky, G. I. Csonka, O. A. Vydrov, G. E. Scuseria, L. A. Constantin, X. Zhou, K. Burke. *Phys. Rev. Lett.* **2008**, *100*, 136406.

13 G. Wulff. *Z. Krist-Cryst. Mater.* **1901**, *34*, 449-530.

14 G. D. Barmparis, Z. Lodziana, N. Lopez, I. N. Remediakis. *Beilstein J. Nanotechnol.* **2015**, *6*, 361-368.

15 J. M. Rahm, P. Erhart. *J. Open Source Softw*. **2020**, *5*, 1944.

16 N. Yang, J. S. Yoo, J. Schumann, P. Bothra, J. A. Singh, E. Valle, F. Abild-Pedersen, J. K. Nørskov, S. F. Bent. *ACS Catal.* **2017**, *7*, 5746-5757.

17 S. S. C. Chuang. Handbook of Climate Change Mitigation. New York: Springer, **2012**, pp 1605-1621.

18 H. T. Luk, C. Mondelli, D. C. Ferré, J. A. Stewart, J. Pérez-Ramírez. *Chem. Soc. Rev.* **2017**, *46*, 1358-1426.

19 G. Wen, Q. Wang, R. Zhang, D. Li, B. Wang. *Chem. Chem. Phys.* **2016**, *18*, 27272-27283.

20 B. Wang, W. Guo, L. Ling, R. Zhang. *Appl. Surf. Sci.* **2019**, *488*, 434-444.

21 C. Wang, J. Zhang, G. Qin, L. Wang, E. Zuidema, Q. Yang, S. Dang, C. Yang, J. Xiao, X. Meng, C. Mesters, F.-S. Xiao. *Chem.* **2020**, *6*, 646-657.

22 C. Wang, Y. Huang, L. Wang, F.-S. Xiao. *Mater. Chem. Front.* **2022**, *6*, 663-679.

23 F. Zhang, W. Zhou, X. Xiong, Y. Wang, K. Cheng, J. Kang, Q. Zhang, Y. Wang. *J. Phys. Chem. C.* **2021**, *125*, 24429-24439.

24 P. Carrillo, R. Shi, K. Teeluck, S. D. Senanayake, M. G. White. *ACS Catal.* **2018**, *8*, 7279-7286.

25 X. Pan, Z. Fan, W. Chen, Y. Ding, H. Luo, X. Bao. *Nat. Mater.* **2007**, *6*, 507-511.

26 Y. Liu, F. Göeltl, I. Ro, M. R. Ball, C. Sener, I. B. Aragão, D. Zanchet, G. W. Huber, M. Mavrikakis, J. A. Dumesic. *ACS Catal.* **2017**, *7*, 4550-4563.

27 F. Li, H. Ma, H. Zhang, W. Ying, D. Fang. Ethanol synthesis from syngas on Mn- and Fe-promoted Rh/γ-Al_2O_3. *C. R. Chimie.* **2014**, *17*, 1109-1115.

28 Y. Liu, K. Murata, M. Inaba, I. Takahara, K. Okabe. *Catal. Today* **2011**, *164*, 308-314.

29 C. Li, J. Liu, W. Gao, Y. Zhao, M. Wei. *Catal. Lett.* **2013**, *143*, 1247-1254.

30 W. Liu, S. Wang, T. Sun, S. Wang. *Catal. Lett.* **2015**, *145*, 1741-1749.

31 D. Mei, R. Rousseau, S. M. Kathmann, V.-A. Glezakou, M. H. Engelhard, W. Jiang, C. Wang, M. A. Gerber, J. F. White, D. J. Stevens. *J. Catal.* **2010**, *271*, 325-342.

32 W. Chen, Y. Ding, X. Song, T. Wang, H. Luo. *Appl. Catal. A-Gen.* **2011**, *407*, 231-237.

33 W. Zhou, S. R. Docherty, E. Lam, C. Ehinger, X. Zhou, Y. Hou, P. Laveille, C. Copéret. *J. Am. Chem. Soc.* **2025,** *147*, 12890-12896.

34 B.Ravel and M. Newville, *J Synchrotron &adiat*, **2005**, 12, 537-541.

35 F. Calle-Vallejo, J. I. Martínez, J. M. García-Lastra, P. Sautet, D. Loffreda. *Angew. Chem. Int. Ed.* **2014**, *53*, 8316-8319.
